# Remembering Who Was Where: A Happy Expression Advantage for Face Identity-Location Binding in Working Memory

**DOI:** 10.1037/xlm0000522

**Published:** 2018-04-19

**Authors:** Sara Spotorno, Megan Evans, Margaret C. Jackson

**Affiliations:** 1 Institute of Neuroscience and Psychology, University of Glasgow, and School of Psychology, University of Aberdeen; 2 School of Psychology, University of Aberdeen

**Keywords:** faces, emotional expression, visuospatial working memory, maintenance, eye movements

## Abstract

It is well established that visual working memory (WM) for face identity is enhanced when faces display threatening versus nonthreatening expressions. During social interaction, it is also important to bind person identity with location information in WM to remember who was where, but we lack a clear understanding of how emotional expression influences this. Here, we conducted two touchscreen experiments to investigate how angry versus happy expressions displayed at encoding influenced the precision with which participants relocated a single neutral test face to its original position. Maintenance interval was manipulated (Experiment 2; 1 s, 3 s, 6 s) to assess durability of binding. In both experiments, relocation accuracy was enhanced when faces were happy versus angry, and this happy benefit endured from 1-s to 6-s maintenance interval. Eye movement measures during encoding showed no convincing effects of oculomotor behavior that could readily explain the happy benefit. However, accuracy in general was improved, and the happy benefit was strongest for the last, most recent face fixated at encoding. Improved, durable binding of who was where in the presence of a happy expression may reflect the importance of prosocial navigation.

During social interaction in our everyday life we use working memory (WM) to continuously keep track of the identity and the position of the people around us to create a coherent and fluid picture of our world. It is also crucial that we are sensitive to the facial expressions of emotion that people convey. Decoding emotional expression and associating its source to a specific person and their specific location in space are essential aspects of our ability to interpret others’ intentions and prepare and execute our own behavior, adapting to the personal context we are acting in. However, we have a very poor understanding of how emotional expression influences our ability to bind person identity and location information in visuospatial WM, and we address this in the current study.

Much research has highlighted the social and biological importance of facial emotional expressions and their strength in guiding attention, WM processes and, consequently, behavior. Considering attentional allocation (e.g., visual search or dot-probe paradigms), the prioritization of emotional versus neutral faces has been well established, but emotion-specific effects are mixed across studies. Focusing on studies using only real faces, some show preferential biases to angry and fearful faces (e.g., Feldmann-Wüstefeld, Schmidt-Daffy, & Schubö, 2011; Fox & Damjanovic, 2006; Pinkham, Griffin, Baron, Sasson, & Gur, 2010), while others report enhanced attention allocation to happy or surprised faces (Becker, Anderson, Mortensen, Neufeld, & Neel, 2011; Juth, Lundqvist, Karlsson, & Ohman, 2005; Williams, Moss, Bradshaw, & Mattingley, 2005). While these results may seem contradictory, it is notable that attentional biases to threatening faces are reported mainly for highly anxious individuals (e.g., Bradley, Mogg, Falla, & Hamilton, 1998; Fox, Russo, Bowles, & Dutton, 2001; Mogg, Millar, & Bradley, 2000), while preferential attentional allocation to positively valenced faces are reported in samples of healthy young individuals (e.g., Becker et al., 2011). Pool and colleagues suggest that attentional biases are driven not necessarily by valence, but by motivational value (Pool, Brosch, Deplanque, & Sander, 2015).

It is essential to note that these tasks used to assess the effect of emotion on attentional selection require little more than detection of faces conveying a given or different emotion. They do not require processing of specific face identity information or any coding of where exactly the emotional signal was located in space. To explore the more complex nature of everyday social interactions, research must take into account the engagement of high-level WM systems representing face identity and spatial sources of emotional information.

A growing number of studies have examined the influence of emotional expression on WM for face identity. Research among healthy young adults has usually used a delayed match-to-sample paradigm, in which participants decide whether a test face presented at retrieval matches in identity one of the faces shown a few seconds earlier. Faces at encoding conveyed emotion but this information was not task-relevant, participants simply had to remember identity. These studies have demonstrated not only enhanced WM for faces showing anger versus a neutral expression (Jackson, Wu, Linden, & Raymond, 2009; see also Sessa, Luria, Gotler, Jolicœur, & Dell’Acqua, 2011, for fearful vs. neutral faces advantage in WM), but also an advantage for faces conveying anger compared with happiness (Jackson, Linden, & Raymond, 2014; Jackson, Wolf, Johnston, & Linden, 2008; Jackson et al., 2009; see also Becker, Mortensen, Anderson, & Sasaki, 2014, and Stiernströmer, Wolgast, & Johansson, 2016). These findings indicate that threat cues boost the allocation of visual WM resources to process person information with greater accuracy, a response that may arise from a basic mechanism aimed at protecting our biological survival, as well as our social and emotional well-being.

Two studies have addressed the question of whether emotional expression can influence spatial WM accuracy for faces in general, with no requirement to encode specific face identity. Using a modified version of the Corsi-block paradigm, Bannerman, Temminck, and Sahraie (2012) asked participants to reproduce in the same order the sequence of locations inhabited by either happy, angry, fearful, or neutral faces. They found no effect of emotion on spatial WM accuracy, and no difference in performance when the locations were signaled by the presence of faces versus a change in luminance. In contrast, Gonzáles-Garrido and colleagues (2013) asked participants to indicate, in inverse order with respect to presentation order, the locations of four or six faces which had been sequentially and randomly presented each in one of six possible screen regions. This is a more demanding WM task requiring manipulation of the encoded information. They found better reverse sequence reports when the locations were occupied by happy faces compared with fearful and neutral faces (Gonzáles-Garrido et al., 2013).

In both of the previous studies, face identity was irrelevant. But in reality, it is often crucial to note *who* we interact with and bind visual identity and spatial location information together. To the best of our knowledge, only one study has examined the effect of emotional expression on WM for who was where. Using a Face Relocation Tasks (FRT), Terburg, Aarts, and van Honk (2012) required participants to replace in their original location a set of eight faces (four happy or angry, plus four neutral counterparts sharing the same identity), which had been presented for encoding simultaneously in random screen locations. During maintenance and retrieval phases empty placeholders remained on screen, into which participants replaced all eight same face images in any order they chose. The authors reported overall a small advantage in relocation accuracy for happy than for angry faces, but individuals with higher trait anxiety scores showed better visuospatial WM for angry faces. Note that in this study, participants were required to relocate each face exactly according to location, identity, and expression properties, thus this task involved triple binding between spatial, visual, and emotional elements.

Although these findings are interesting, there are some issues with this particular FRT paradigm which may impede our understanding of exactly how emotion influences face identity-location binding. First, relocating multiple faces, or requiring to retrieve a sequence of positions, introduces different maintenance intervals between the end of the encoding phase and the start of the retrieval phase for each face. This then may vary the degree to which face representations decay or degrade in WM over time, and also the amount of potential perceptual and mnemonic interference from the previously relocated faces in that given trial. Second, the number of possible relocation placements is not equivalent for each face during the course of a trial, because it decreases as the number of previously relocated faces increases. Third, the data reported do not allow us to understand whether participants chose to relocate the emotional (happy/angry) faces first or the neutral counterparts (or in a more random order), and how this may reflect the influence of emotion on relocation accuracy. Fourth, in each encoding display there were four face identities shown twice simultaneously, one in expressive form and the other in neutral form, which introduces a potential confound of identity repetition and reduces the validity of the design. Finally, study and test faces were identical images, thus it remains unclear whether, or in what portion, the results arise simply from low-level feature matching or higher level processes involving identity recognition and identity binding to location.

Another aspect that has to be taken into account in examining visuospatial WM is that the traditional approach of assessing performance exclusively in terms of “all-or-none” correct or incorrect responses may be too rudimentary. Bannerman et al. (2012), Gonzáles-Garrido et al. (2013), and Terburg et al. (2012) used placeholders during maintenance and retrieval and thus measured relatively coarse ability to relocate information. Such an assessment does not reflect the fact that memory may be not as simple as “remember” or “forget” but better defined in terms of more subtle variations in the visuospatial precision of representations (see Bays, Catalao, & Husain, 2009, or Fukuda, Awh, & Vogel, 2010). Real life does not provide external placeholders, and instead we rely on an internal map of our environment. To analyze the finer-grained quality of the visuospatial WM representation, we need to measure the precision of relocation as a continuum. Pertzov and colleagues did just this, and measured the precision of identity-location bindings for complex everyday objects and fractals in WM (Pertzov, Dong, Peich, & Husain, 2012). These authors presented for encoding an array of between one and five objects on a touchscreen. After a 1,000-ms blank retention period, all the objects reappeared simultaneously but in different locations and participants were asked to touch and drag each one back to its original place (no place holders were used). In further experiments participants relocated one of two items that were shown at test (one target from the encoding display plus one foil) that they thought had been present on that trial. The authors measured both a binomial and a continuous measure of spatial relocation accuracy by computing, respectively, (1) whether or not objects were placed within 5 degrees of the original location (yes/no, coarse accuracy), and (2) for objects placed within the 5-degree radius threshold, they computed the absolute distance between the original and the reported placements in degrees of visual angle (continuous measure, finer precision; see also Pertzov, Avidan, & Zohary, 2009). In addition, they analyzed “swap” errors—when an object was relocated within the 5-degree radius region of another one—which specifically enabled investigation of identity-location binding failures. They found that increasing WM load and lengthening maintenance interval decreased the spatial accuracy with which participants relocated the objects. They also measured eye movements during encoding and showed that the last item to be fixated was better relocated than other items, a form of recency effect.

In the present work, we sought to examine how emotional expression influences the binding of who was where in visuospatial WM for emotional faces, utilizing an adaptation of the paradigm and response measures of Pertzov et al. (2012), while improving upon the methodological issues of the Face Relocation Task outlined previously. Specifically, the study faces at encoding were of different identities (no repeats per display) and were always all happy or all angry to create a homogenous display in which there was no potential for attentional bias to one emotion over another. While faces at encoding were always expressive, at retrieval there was only one test face which was always neutral. Changing the expression to neutral at test ensures that relocation performance is anchored to the binding of face identity with spatial location, rather than image location matching, and that the maintenance interval between encoding and retrieval is constant across all trials and emotion conditions.

Here we report the results of two experiments. In Experiment 1, WM load was manipulated and there were one, two, three, or four happy or angry faces presented for encoding at pseudorandom screen locations. After a 1,000-ms blank maintenance interval, a single neutrally expressive face was shown in the center of the screen, and participants were required to touch and drag this face back to its original position as precisely as they could. In Experiment 2, WM load was held constant—four faces were presented for encoding—but we varied maintenance interval (1 s, 3 s, 6 s) to examine the durability of any emotion effects on visuospatial binding over time.

We analysed the influence of emotional expression on coarse accuracy, fine precision, and swap errors. The threat advantage shown for angry faces in previous studies, which measured WM for face identity in the absence of location constraints (e.g., Jackson et al., 2014), may predict that the presence of anger at encoding will strengthen the binding of identity to location. One could argue that it is important to encode and maintain the source of threat in WM more accurately than the source of a more benign social signal such as a smiling person. This would be reflected in improved accuracy and precision. However, the happy advantage found by Terburg et al. (2012) using the multiple face relocation task suggests that accurate person identity-location binding may favor more positive social signals, at least among individuals who do not display high anxiety traits. Using our improved paradigm with healthy young adults, we also measured social anxiety, levels of autistic-like traits, and current mood state to explore the potential influence of emotional expression on face identity-location binding in WM in more depth. In addition, we recorded oculomotor behavior during encoding to examine the influence of overt attentional selection on WM performance. We move our eyes on average three to four times per second. Eye movements enable our visual system to scrutinize small portions of the visual field in high detail by placing them in the high-acuity central area of the retina (i.e., the fovea), while the other regions of the visual field failing outside the fovea are perceived with decreased accuracy as retinal eccentricity increases (see Findlay & Gilchrist, 2003). In our everyday life, our visuospatial attention is thus tightly coupled with the position of our gaze. As a consequence, analyzing how we select information with the eyes during encoding does, on the one hand, take into account what we usually and spontaneously do to extract and memorize information from faces and, on the other hand, is a powerful means with which to study the fine mechanisms and temporal dynamics of information uptake. Here we were specifically interested in the mechanisms and dynamics that may be modulated by the emotional content of the faces, and how this may modulate visuospatial WM.

## Method

### Participants

All participants had normal or corrected-to-normal vision, and took part in the study for course credits or were reimbursed £5 for their time. In Experiment 1, 48 healthy individuals (18 males), aged 19–36 (*M* = 24, *SD* = 4), took part. Forty-six were right-handed and two were ambidextrous (overall participants’ LQ: *M* = 73, *SD* = 21). In Experiment 2, 48 healthy individuals (7 males), aged 19–38 (*M* = 22, *SD* = 4), took part. Forty-four were right-handed, three were ambidextrous and one was left-handed (overall participants’ LQ: *M* = 72, *SD* = 31). Handedness was measured in terms of Laterality Quotient (LQ) at the Edinburgh Laterality Inventory (ELI; Oldfield, 1971; paper-and-pen version). The study was approved by the local Ethics Committee in the School of Psychology at the University of Aberdeen, and carried out in accordance with the British Psychology Society guidelines. All participants provided written informed consent prior to participating and were fully debriefed at the end.

### Materials and Apparatus

Twenty-four facial images in total, comprising eight male identities conveying angry, happy or neutral expressions, were used as stimuli. They were taken from the Radboud database (Langner et al., 2010) and modified with Adobe Photoshop Creative Suite (CS; Adobe, San Jose, CA), so that each image was grayscale transformed, had nonfacial features removed and subtended an oval of 100 × 140 pixels (2.63 × 3.68 cm, 3.70 × 5.19 degree of visual angle). The faces were presented on a white background at both encoding and retrieval. Face location at encoding was randomly generated in Matlab, 2012b (MathWorks, Inc., Natick, MA) within the screen’s presentation area, but with the following constraints: A minimum distance of 7 degrees between the edge of the face and the center of the screen, and a minimum distance of 14 degrees between the centers of two faces. This latter constraint was imposed to prevent spatial uncertainty as a result of crowding (see Levi, 2008), and to create a “safe area” critical for analysis of WM performance (see Data Handling and Analysis.) A given combination of placements was presented only once for each participant, but the same placements or their left/right reversal (see Procedure) were used for all participants, across all versions of the experiment (different random locations were generated for Experiments 1 and 2). We limited overall stimulus presentation to a central area of the screen sized 1,120 × 706 pixels, 29.69 × 18.72 cm, 42.53 × 26.81 degrees, to ensure that participants could reach and touch all areas comfortably from their seating position behind the tower-mounted eye-tracker.

Moreover, a given face identity was randomly attributed to a given random location, but we made sure that, for each participant and across all participants, each of the eight identities appeared almost an equal number of times in the encoding display (maximum difference between the most presented and the least presented face = 0.12% of total trials), for each level of load and for each emotion, as a test face or as another copresent face (for Loads 2 to 4). In addition, by mirror-reversing the placements of the faces between participants (see Materials), we counterbalanced the side of the screen in which each face identity was presented at encoding, for each load and for each emotion. The side the tested face came from at encoding was also counterbalanced within participants, to control for any difference in relocation precision in the ipsilateral versus contralateral side of the screen with respect to the hand, because of potential difficulties related to the arm or hand movement, or to spatial compatibility aspects (see Proctor & Reeve, 1989).

We used the Negative Affect Schedule (PANAS; Watson, Clark, & Tellegen, 1988) to measure natural mood state (at the start), the Liebowitz Social Anxiety Scale (LSAS; Mennin et al., 2002) to measure social anxiety, and the Autism-Spectrum Quotient (AQ; Baron-Cohen, Wheelwright, Skinner, Martin, & Clubley, 2001) to measure autistic-like traits. The questionnaires were presented on the screen using E-Prime 2.0 (Psychology Software Tools, Pittsburgh, PA), and participants were informed that they were free to skip any questions to which they did not want to respond.

The WM experiments were generated in Experiment Builder (SR Research, Canada). They were conducted on a Dell Optiplex 7010 computer running Windows 7. Stimuli were shown on an EliteOne 800 touchscreen (50.9 × 28.7 cm, 72.91 × 41.01 deg), with a resolution of 1920 × 1080 pixels and a refresh rate of 60 Hz. A chin rest stabilized the eyes 40 cm away from the screen. Eye movements were recorded using a tower-mounted EyeLink 1000 Plus at a sampling rate of 1000 Hz (SR Research, Canada). Viewing was binocular, but only the dominant eye (left eye for 12 participants) was tracked.

### Procedure

**Figure 1 fig1:**
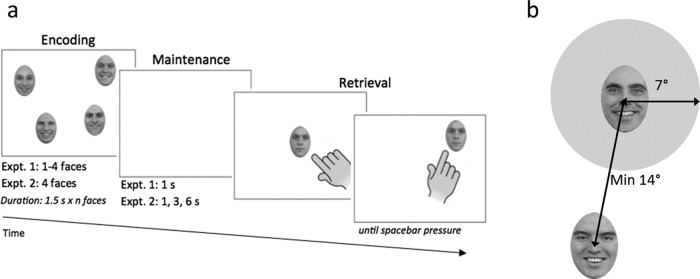
(a) Illustration of trial example, (b) illustration of the “safe zone” used to measure accuracy and precision.

The experiments were conducted in a dimly illuminated room in which participants were seated in front of the computer screen. Prior to either experiment, participants were given the PANAS and the laterality questionnaire. Social anxiety and autistic-like traits were measured on completion of the main WM task. Prior to the WM experimental task, each participant underwent a randomized nine-point calibration and validation procedure. Recalibrations were performed during the task if necessary. Before each trial a single-point calibration check was applied as the participant fixated a dot in the center of a white background. This was follow by a central fixation cross, over a white background, presented for 500 ms, and then by the encoding display. In Experiment 1 the encoding display contained one to four faces while only 4 faces were displayed in Experiment 2. In both experiments, all faces at encoding displayed an angry expression or a happy expression, and were presented simultaneously for 1.5 s × Number of faces. A maintenance interval followed the encoding faces offset, comprising a blank (white) display presented for 1 s (Experiment 1) or 1 s/3 s/6 s randomized in Experiment 2. Following the maintenance interval, a single neutrally expressive face was presented in the center of a white background and always matched the identity of one of the faces shown at encoding (Figure 1a for a trial example). As the faces at encoding were presented at least at 7 degrees from the center of the screen (see Materials and Apparatus), there was never any spatial overlap with the test face. Participants had to touch and drag the test face back to its original location using their right index finger. They were told to be as precise as possible, with no time limit. They were allowed to change the relocation placement as many times as they wished. When they placed the face in the location of their final choice, they pressed the spacebar on the computer keyboard to end the trial. All the experimental factors were counterbalanced within and between participants.[Fig-anchor fig1]

In Experiment 1, each participant was presented with 256 trials (50% angry, 50% happy), and there were 32 angry and happy trials per load condition. Sixteen practice trials preceded the main experiment. In Experiment 2, each participant was presented with 192 trials (50% angry, 50% happy) and there were 32 trials per maintenance interval condition. Twelve practice trials preceded the main experiment. Emotion and load/maintenance conditions were randomized. In both experiments, there were 12 versions of the experiment to counterbalance the experimental factors, the facial identity of the test face, the number of times each face identity was presented in a given experimental condition, and the mirror-reversing of face placement along the horizontal axis of the screen to control for presentation side. Four participants were tested in each version per experiment. The WM experiment lasted for about one hour and the whole session was approximately 90 minutes in total. Frequent breaks were provided to aid fatigue. Calibration and validation procedures were performed at the end of each break.

### Data Handling and Analysis

#### Data exclusions

We discarded from all analyses trials in which the average calibration error was ≥0.5 degrees or the maximum error in one of the calibration points was ≥1 degree (% of total data: 0.7% in Experiment 1 and 0.9% in Experiment 2), or the error in the single point calibration check before trial start was ≥1 degree (% of remaining data: 1.4% in Experiment 1 and 0.3% in Experiment 2). We then discarded the trials in which participants were fixating outside the 7-degree-radius area from the center of the screen, that corresponded to imposed minimum distance between face’s edge and screen center (see Procedure): This removed a further 2.3% of data in Experiment 1 and 4.3% of data in Experiment 2. We finally discarded trials in which the test face was not fixated during encoding (% of remaining data: 1.8% in Experiment 1 and 0.7% in Experiment 2). In total, 6.0% of data (742 trials) in Experiment 1 and 7.6% of data (665 trials) in Experiment 2 were removed prior to analysis as a result of these removals.

#### WM performance data

WM performance was examined using three types of data: accuracy, precision, and swap errors.

##### Accuracy

A 7-degree “safe area” was created around each of the encoding face locations, from the center of each face (Figure 1b). This distance corresponded to half of the minimum distance between the centers of two faces in the encoding display. A test face relocated within this safe area was coded as a “correct” response, and a test face relocated beyond this area was coded as an “incorrect” response.

##### Precision

For all correct responses, we measured how precisely the test face was repositioned within the safe area, using the absolute distance in degrees of visual angle from the center of the test face to the center of where the original face was. Following inspection of the distribution, the distance of relocation was log-transformed to meet assumptions of the modeling analysis we applied to the data (see Linear mixed models section below).

##### Swap errors

For all incorrect responses, we measured the proportion of trials in which the test face was erroneously placed in the location of another face (mis-binding error) compared with a location that had not previously been inhabited (random error). For mis-binding errors, we also computed how precisely the test face was positioned within the wrong face location, using the 7-degree-radius safe area of the non-test face.

#### Linear mixed models

Linear Mixed Models (LMMs) were used for the continuous precision data and Generalized Linear Mixed Models (GLMMs) were used for the binomial accuracy data, using the lmer() or glmer() function of the lme4 package (Bates, Maechler, Bolker, & Walker, 2014) in the R programming environment (The R Foundation for Statistical Computing, Version 3.0.3, 2014). LMMs and GLMMs have many advantages over traditional analysis of variance (ANOVA) models. Crucially, they optimize power of the experimental design by performing item analysis and allow a simultaneous estimation of between-subjects and between-item variance. In addition, they are known to be more robust than ANOVAs when a design is not fully balanced as a result of data exclusions (see Kliegl, Masson, & Richter, 2010). In all analyses, only *p* values below the .05 threshold were considered noteworthy, and any effects where *p* > .05 were classed as non-significant. Trends where *p* = .05 - .09 were not considered.

Participants and trials were specified as random factors. For each experiment, the same analyses were run on both accuracy and precision data. Where possible, random slope models were used with maximal random effects structure (Barr, Levy, Scheepers, & Tily, 2013) but without correlations between random slopes and intercepts. When the full model did not converge, we simplified the model using a stepwise procedure. We first removed the slope of the highest order interaction between the fixed effects, and in this way, we gradually reduced model complexity until the model converged. We also simplified the trial term before simplifying the subject term. We always report the most complex model that converged (see Appendix in the online supplemental material for a full list of the structures of the models reported).

For each model, we report the predictors’ coefficients (β values), the *SE* values, the *t* values, or the *z* values for binomial data, and the associated *p* values. In the case of *t* values, associated *p* values are not directly supplied by lme4 package, but were generated using the lmerTest library (Kuznetsova, Bruun Brockhoff, & Haubo Bojesen Christensen, 2016). When an interaction was significant, we ran follow up models to explore it. Graphics were created using the ggplot2 package (Wickham, 2009).

In Experiment 1, we did not include Load 1 in the analyses of performance because there is no identity-location binding processes to assess here—only location memory is engaged. But Load 1 data are a useful baseline to determine whether there was any influence of emotion on relocating the face in general, and to check participants’ raw spatial abilities overall. Relocation response was incorrect on only three trials at Load 1, and overall mean precision of accurate responses at Load 1 was 1.46 degree (*SD* = 0.93), without any significant difference because of emotion (angry faces: *M* = 1.48, *SD* = 0.95; happy faces: *M* = 1.44, *SD* = 0.92; *p* = .427; Model 1.2.2 - see supplementary material for models.), indicating the expected high performance in this easiest condition.

In Experiment 1, the critical GLMMs/LMMs on WM performance included the following predictors: emotion (angry, happy), load coded as sliding contrasts (2 vs. 3, 3 vs. 4), and their interactions. In Experiment 2, the critical GLMMs/LMMs on WM performance included the following predictors: emotion (angry, happy), maintenance interval (MI) coded as sliding contrasts (1 s vs. 3 s, 3 s vs. 6 s), and their interactions.

Further models were run in both experiments. To examine whether relocation distance from original (test or non-test) face position differed between correct responses and swap errors, we also included the type of response (correct, swap error), and its interactions with emotion and load (Experiment 1) or with emotion and maintenance interval (Experiment 2); we will only report for these models the effects involving the type of response. Moreover, to study any modulation of accuracy and precision by individual differences, we analyzed, in separate models: the Autism Quotient (Baron-Cohen et al., 2001), the Liebowitz total score (Mennin et al., 2002), the PANAS positive score and the PANAS negative score (Watson et al., 1988). Each of these predictors was included in a model together with emotion and load (Experiment 1) or with emotion and maintenance interval (Experiment 2), and the possible two-way interactions between predictors; we report for these models only the effects involving the individual measures considered.

#### Oculomotor behavior

We analyzed participants’ eye movements during encoding. Eye-movement raw data were parsed into saccades and fixations using the SR Research algorithm. The regions of interest (ROIs) for scoring eye movements were defined in Matlab, 2012b (MathWorks,Inc., Natick, MA) as a smallest fitted rectangle that encompassed a face. A fixation was considered as being on a specific ROI if the center of gaze indicated by the eye tracker fell within the boundary of the ROI or within 0.5 degree from it. Note that in Experiment 1 we extracted eye-movement data from Load 4 only, because this provided the most opportunity to examine a diverse range of eye-movement patterns, and also allowed us to compare effects with Experiment 2 in which four faces were used in each trial.

Two forms of investigation were carried out on eye movement data.

(1) *Emotion effects on encoding behavior*. This allowed us to assess whether oculomotor behavior during encoding was different when participants viewed a display of happy versus angry faces. We report the effects of emotional expression on (a) mean fixation duration per face and (b) mean visit duration per face. It is useful to look at these two different measures as they can tell us different things about information gathering. Mean fixation duration provides information on how the total time spent on a face was parsed into fixations. But with a single fixation, participants are likely to have been able to gather information from about half of the face area, which means that samples of information obtained from one fixation on a face would overlap with subsequent fixations. Mean visit duration provides a more complete picture of information gathering, as it includes all fixations on a face before the participant directed the gaze away. Mean visit duration therefore indicates how long a participant spent on average looking at a face before moving to another face (or before display offset). After inspection of the distributions, mean fixation duration was log-transformed to meet LMM assumptions. Fixations starting before display onset or ending after display offset were excluded from these analyses. The LMMs carried out included only emotion as a predictor.

(2) *Relationship between oculomotor behavior on tested face and WM performance*. We assessed this to determine whether and how oculomotor behavior at encoding on the face that would then be tested (which was unbeknownst to participants) influenced subsequent relocation accuracy and precision at retrieval. We report five different oculomotor measures here: (a) total fixation time; (b) mean fixation duration; (c) mean visit duration; (d) selection order (the order in which the face to be tested was first fixated in the encoding display, compared with the other faces—primacy effects), and (e) exit order (the order in which the face to be tested was last fixated in the encoding display, compared with the other faces—recency effects). Total fixation time is useful here as it indicates the total time spent gathering information from the test face, and may logically impact on the strength of the WM representation in terms of the amount and quality of information encoded. But it does not tell us much about the sampling strategy of the viewer, which is why we also examined mean fixation duration and mean visit duration on the test face. Following inspection of the distribution and residuals, mean fixation duration was log-transformed to meet LMM assumptions. All these measures were specifically locked to the test face, and included as predictors in the GLMMs/LMMs (each in a different model) together with emotion and their interactions. In Experiment 2, these models were carried out separately for each maintenance interval. For all these models we report only effects involving the oculomotor predictor considered.

To examine primacy effects, selection order was simple contrast coded, comparing when the test face was initially fixated as the first at encoding to each other case in which it was initially fixated in another order (i.e., first vs. second, first vs. third, first vs. last). To examine recency effects, exit order was simple contrast coded, comparing when the test face was exited (fixated for the last time) as the last (fourth) face at encoding to each other case in which it was exited in another order (i.e., last vs. third, last vs. second, last vs. first). For all the analyses of eye-movement behavior concerning selection or exit order of the tested face, we removed the trials in which not all the faces presented at encoding were fixated and those in which there was only a single entry per face, as in this case selection and exit were the same (Experiment 1: 4.4% and 1.4%, respectively, of Load 4 data used in simple WM performance analysis; Experiment 2: 2.2% and 3.7%, respectively, of all data used in simple WM performance analysis). Note that Kendall’s tau rank correlation tests revealed that selection and exit order were not significantly correlated, taking into account either all trials (Experiment 1: τ = 0.008, *p* = .631; Experiment 2, considering each MI separately: maximum *τ =* −0.022, minimum *p* = .0147) or only correct trials (Experiment 1: τ = 0.001, *p* = .948; Experiment 2: maximum *τ =* −0.010, minimum *p* = .573). Models used are listed in the online supplemental material and referred to here by number for reference.

## Results

We present the results for both experiments first in terms of behavioral effects and second in terms of oculomotor activity.

### Behavioral: WM Performance

#### Experiment 1

##### Accuracy

**Figure 2 fig2:**
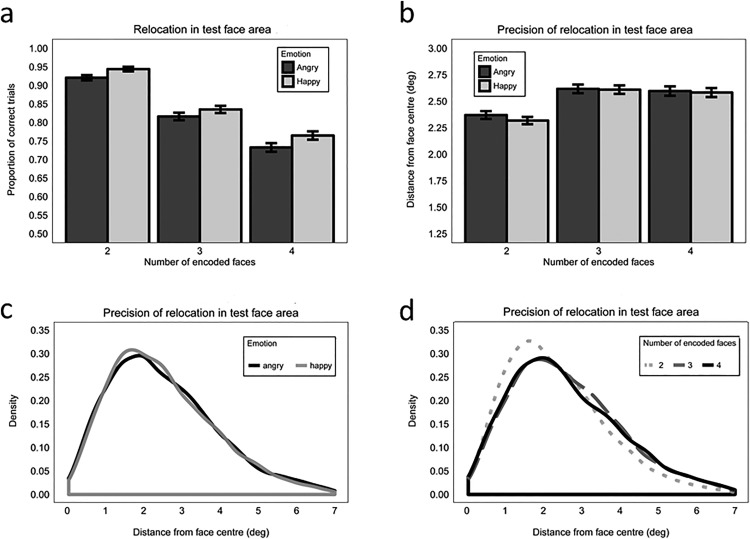
Behavioral results from Experiment 1: (a) accuracy as a function of emotion and load, (b) precision in degrees of visual angle as a function of emotion and load, (c) precision density plot as a function of emotion, (d) precision density plot as a function of load.

Emotional expression influenced the accuracy with which participants positioned the neutrally expressive test face within an area of 7-degree-radius from the center of its original location (i.e., within the “safe area”; Model 1.1.1). A main effect of emotion showed that accuracy was better when faces showed a happy (*M* = 0.85, *SE* = 0.005) versus an angry (*M* = 0.82, *SE* = 0.006) expression at encoding (β = 0.252, *SE* = .084, *z* = 2.99, *p* = .003), see Figure 2a. As expected, accuracy decreased as load increased (Load 2 vs. 3: β = 1.375, *SE* = 0.246, *z* = 5.58, *p* < .001; Load 3 vs. 4: β = 0.717, *SE* = 0.174, *z* = 4.13, *p* < .001). Emotion and load did not interact (maximum *z* = 1.20, minimum *p* = .231).[Fig-anchor fig2]

##### Precision

Emotional expression did not influence the precision with which participants positioned the test face within the safe area of the correct location (*β =*−0.003, *SE* = −0.007, *t* = −0.37, *p* = .71; Model 1.2.1; Figures 2b and 2c). Precision decreased as load increased from 2 to 3 encoded faces (β = 0.052, *SE* = 0.014, *t* = 3.71, *p* < .001), but there was no further decrement in precision when load increased from 3 to 4 faces (β = −0.009 *SE* = 0.014, *t* = −0.58, *p* = .56; Figures 2b and 2d). Emotion and load did not interact (maximum *t* = 0.536, minimum *p* = .594). We also analyzed precision without the constraint of the 7-degree-radius “safe area”, but only considering whether relocation of the test face was closer to the original position of that face at encoding than to one of the other faces, and we found the same pattern of results (load effect two vs. three faces: β = 0.055, *SE* = 0.015, *t* = 3.78, *p* = .001; all other effects: maximum *t* = 0.65, minimum *p* = .520).

##### Swap errors

**Figure 3 fig3:**
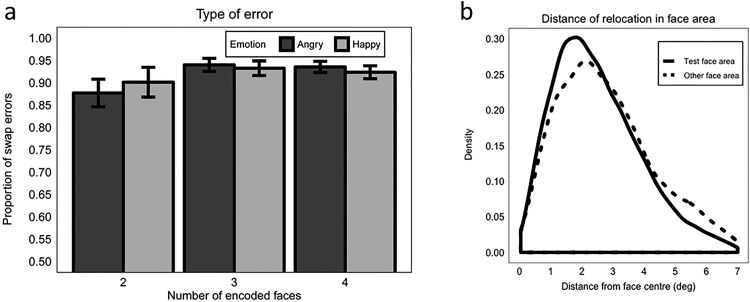
Swap errors from Experiment 1: (a) proportion of swap errors as a function of emotion and load, (b) precision for test face (correct relocation) versus other face (swapped, incorrect relocation).

Overall, 92.64% of errors were swap (i.e., mis-binding) errors in which the test face was positioned in the 7-degree-radius location of the wrong face. This indicates that participants were much more likely to make swap errors than to make random errors. The proportion of swap errors was not significantly modulated by emotion (*β =* −0.120, *SE* = 0.026, *z* = −0.47, *p* = .638; Model 1.3). Moreover, even though there were more opportunities to make a swap error as the number of possible erroneous regions to choose from increased, the proportion of swap errors did not increase as load increased (Load 2 vs. 3: β = −0.264, *SE* = −0.615, *z* = −0.43, *p* = .667; Load 3 vs. 4: β = −0.049, *SE* = −0.453, *z* = −0.11, *p* = .913). Emotion and load did not interact (maximum *z* = 0.27, maximum *p* = .784; Figure 3a).[Fig-anchor fig3]

We also analyzed whether the precision of relocation differed between correct responses (i.e., relocations in the “safe area” of the test face) and swap errors, and also whether the precision in case of mis-binding was influenced by emotion or load (Model 1.4). We found that precision tended to be better when participants retained the correct identity-binding information (*M* = 2.51 degree, *SE* = 0.016) compared with when they made swap errors (*M* = 2.77 deg, *SE* = 0.042), but this did not reach significance (β = −0.023, *SE* = 0.012, *t* = −1.86, *p* = .066; Figure 3b). The type of response (correct vs. swap error) did not interact with either emotion or load (maximum *t* = 0.851, minimum *p* = .398). Therefore, emotion did not modulate relocation precision within the incorrect non-test face location, or within the correctly located region.

##### Individual differences

There were no significant effects of autistic-like traits, social anxiety, or mood state on accuracy and precision, regardless of load or emotion (all *p*s > .061; Models 2).

#### Experiment 2

##### Accuracy

Accuracy was significantly better when faces showed a happy (*M* = 0.77, *SE* = 0.011) versus an angry (*M* = 0.73, *SE* = 0.010) expression at encoding (β = 0.338, *SE* = 0.076, *z* = 4.36, *p* < .001; Model 3.1). We examined specifically load 4 data from Experiment 1 and we found a significant happy benefit of a comparative nature (happy faces: *M* = 0.76, *SE* = 0.011; angry faces: *M* = 0.73, *SE* = 0.012; β = 0.201, *SE* = 0.092, *z* = 2.19, *p* = .028; Model 1.1.2).

**Figure 4 fig4:**
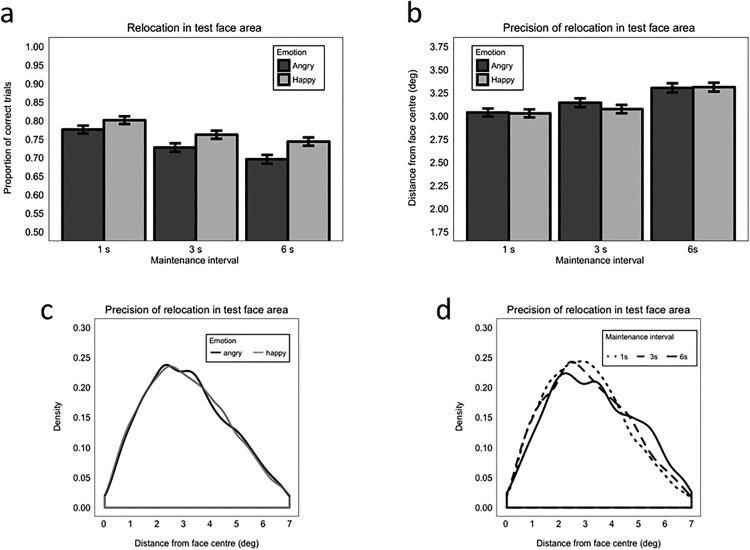
Behavioral results from Experiment 2: (a) accuracy as a function of emotion and maintenance interval, (b) precision in degrees of visual angle as a function of emotion and maintenance interval, (c) precision density plot as a function of emotion, (d) precision density plot as a function of maintenance interval.

Accuracy decreased as maintenance interval (MI) increased (MI 1 s vs. 3 s: β = −0.281, *SE* = 0.079, *z* = −3.55, *p* < .001; MI 3 s vs. 6 s: β = −0.191, *SE* = 0.073, *z* = −2.63, *p* = .009). Emotion did not interact with maintenance interval (maximum *z* = 0.36, minimum *p* = .716; Figure 4a).[Fig-anchor fig4]

##### Precision

There was no influence of emotional expression on the precision with which participants accurately relocated the test face (*β = −0.003, SE* = 0.008, *t* = −0.36, *p* = .717; Model 3.2; Figures 4b and 4c). Precision did not significantly differ between the 1 s and 3 s maintenance interval (β = 0.011, *SE* = 0.008, *t* = 1.38, *p* = .171), but it did significantly decrease as the maintenance interval increased from 3 s to 6 s (β = 0.028, *SE* = 0.009, *t* = 3.01, *p* = .004; Figure 4b and 4d). There was no interaction between emotion and maintenance interval (maximum *t* = 0.54, minimum *p* = .592). Note that, as in Experiment 1, we found a similar pattern of results when we analyzed precision without the constraint of the 7-degree-radius safe area, with the only exception that the decrease in precision between 1 s and 3 s maintenance interval became significant (β = 0.017, *SE* = 0.008, *t* = 2.13, *p* = .034).

##### Swap errors

**Figure 5 fig5:**
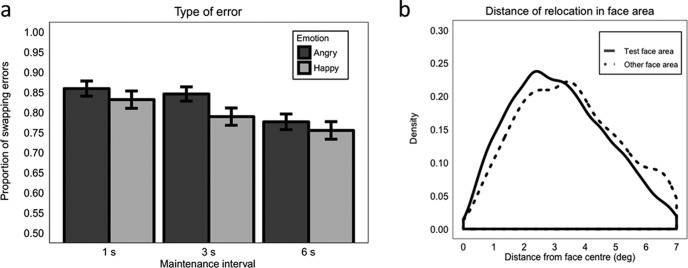
Swap errors from Experiment 2: (a) proportion of swap errors as a function of emotion and maintenance interval, (b) precision for test face (correct relocation) versus other face (swapped, incorrect relocation).

Overall, 80.73% of errors were swap errors in which the test face was positioned in the location of the wrong face, indicating that the majority of errors were because of mis-binding in the WM representation of identity and position information, rather than a random response. A higher proportion of swap versus random errors were made when faces were angry (*M* = 0.82, *SE* = 0.010) than happy (*M* = 0.79, *SE* = 0.010), but this did not reach significance (β = 0.243, *SE* = 0.129, *z* = 1.89, *p* = .058). While no significant difference was found comparing 1 s to 3 s maintenance interval (β = 0.201, *SE* = 0.197, *z* = 1.02, *p* = .308), the proportion of swap errors significantly decreased as the maintenance interval increased from 3 s to 6 s (β = 0.520, *SE* = 0.182, *z* = 2.86, *p* = .004; Figure 5a). This shows that participants were more likely to make more random errors at the longest maintenance interval. Emotion and maintenance interval did not interact (maximum *z* = 1.09, minimum *p* = .275; Model 3.3).[Fig-anchor fig5]

As in Experiment 1, we analyzed whether the precision of relocation differed between correct responses and swap errors (Model 3.4). We also analyzed whether the precision of swap errors was influenced by emotion or maintenance interval. Precision was significantly better for the correct relocation of the test face (*M* = 3.15 deg, *SE* = 0.019) versus incorrect placing of the test face in a (7-degree radius) location originally inhabited by a non-test face (*M* = 3.49 degrees, *SE* = 0.038; β = 0.197, *SE* = 0.061, *t* = 3.27, *p* = .002; Figure 5b). Neither emotion nor maintenance interval modulated precision in the case of mis-binding (no interaction involving the type of response: maximum *t* = 1.59, minimum *p* = .112).

##### Individual differences

As in Experiment 1, there were no significant effects of autistic-like traits, social anxiety, or mood state on accuracy or precision, regardless of maintenance interval or emotion (all *p*s > .075; Models 4).

### Results Summary: Behavioral Data

In both Experiments 1 and 2, accuracy of test face relocation within 7-degrees from the center of its original location was significantly better when faces were happy versus angry at encoding. In Experiment 1, this effect of emotion was not modulated by load and, in Experiment 2, it was not modulated by maintenance interval. However, overall accuracy decreased as load and maintenance interval increased in Experiments 1 and 2, respectively. Neither experiment showed an influence of emotion on the precision with which the test face was relocated within this 7-degree area. However, both load and maintenance interval modulated precision. In Experiment 1, precision decreased as load increased from two to three faces but there was no further decline in precision from three to four faces. In Experiment 2, precision did not decline between 1 s and 3 s, but showed a significant decline from 3 s to 6 s maintenance interval.

Across experiments, the majority of relocation errors were swap errors (i.e., mis-binding errors) where the test face was erroneously placed in the location of a different face (92.64% and 80.73% in Experiments 1 and 2, respectively). The proportion of swap errors was not significantly modulated by emotion in either Experiment 1 or 2, or by load in Experiment 1. However, the proportion of swap errors decreased as maintenance interval increased from 3 s to 6 s (Experiment 2), indicating that location errors became more random as maintenance demands increased. This shows that, although there were more broad-scale repositioning errors for angry faces overall (reflected in lower accuracy), these errors were not statistically different in nature to those made when happy faces were incorrectly repositioned. We did not make any specific predictions on how emotional expression may influence error type, and the number of error trials within these analyses is small, so this is not discussed further.

Finally, precision was significantly better when the test face was positioned correctly (i.e., when participants had retained the correct identity-location binding information) versus when it was incorrectly placed in the location of a different face (i.e., when mis-binding had occurred). There was no impact on performance from individual differences related to autistic-like traits, social anxiety, or current mood state.

### Oculomotor Activity

As described in Data Handling and Analysis, the following sections concerning Experiment 1 take into account only trials with four faces presented at encoding.

#### Emotion effects on encoding behavior

We analyzed whether the emotion conveyed by the encoded faces modulated oculomotor inspection behavior in terms of the mean duration of each fixation or each visit per face.

##### Experiment 1

Emotion did not significantly affect either mean fixation duration per face (β = −0.003, *SE* = 0.003, *t* = −0.87, *p* = .393; angry faces: *M* = 319 ms, *SE* = 2.11; happy faces: *M* = 317 ms, *SE* = 2.16; Model 5.1) or mean entry duration per face (β = −0.006, *SE* = 0.004, *t* = −1.43, *p* = .157; angry faces: *M* = 631 ms, *SE* = 6.07; happy faces: *M* = 624 ms, *SE* = 6.07; Model 5.2).

##### Experiment 2

Mean fixation duration per face was slightly but significantly longer when the encoding display comprised angry faces (*M* = 310 ms, *SE* = 1.13) compared with happy faces (*M* = 306 ms, *SE* = 1.04; β = −0.004, *SE* = 0.002, *t* = −2.27, *p* = .027; Model 6.1.1). To investigate whether this difference was related to WM accuracy at retrieval, we ran a further model (Model 6.1.2) considering emotion, accuracy, and their interaction as predictors. We found that, beyond the effect described above (longer mean fixation duration in angry than happy displays), no other effects were significant (maximum *t* = −1.56, minimum *p* = .121). This suggests that the influence of emotion on mean fixation duration was not related to WM performance. (We did not run this same model on precision data because of the absence of an effect of emotion on precision.) In contrast, mean visit duration per face was not significantly affected by emotion (angry faces: *M* = 621 ms, *SE* = 3.01; happy faces: *M* = 615 ms, *SE* = 2.89; β = −0.003, *SE* = 0.003, *t* = −1.30, *p* = .201; Model 6.2).

#### Relationship between oculomotor behavior on tested face at encoding and WM performance

##### Experiment 1: Accuracy

###### Continuous eye-movement predictors

We first examined whether total fixation time (Model 7.1), mean fixation duration (Model 7.2), and mean visit duration (Model 7.3) on the test face (the face at encoding that was subsequently presented for relocation) during the encoding display predicted accuracy of relocation of that face within its 7-degree-radius safe area. No oculomotor measures significantly predicted WM accuracy during relocation, regardless of the emotion conveyed at encoding (all *z*s < 1.89, all *p*s > .059).

###### Selection order

No effects involving selection order were significant (maximum *z* = 1.14, minimum *p* = .256), indicating that accuracy of relocation did not depend on whether the test face during encoding was selected first, second, third, or fourth, and this was regardless of the emotional expression conveyed (Model 7.4).

###### Exit order

**Figure 6 fig6:**
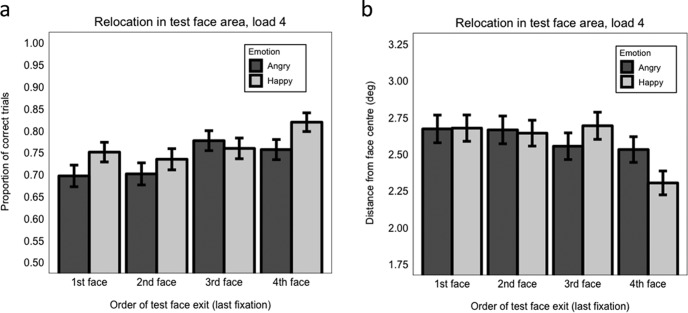
(a) accuracy and (b) precision as a function of exit fixation order in Experiment 1, load 4 data only.

Accuracy was significantly enhanced when the test face was the last face exited compared with when it was exited second (β = −0.605, *SE* = 0.187, *z* = −3.24, *p* = .001) and first (β = −0.581, *SE* = 0.172, *z* = −3.37, *p* < .001). There was no significant difference in accuracy between last and third (penultimate) exits (β = −0.201, *SE* = 0.203, *z* = −0.99, *p* = .321). However, the interaction between emotion and exit order last versus third was significant (β = −0.591, *SE* = 0.299, *z* = −1.98, *p* = .048). Follow-up analyses examining separately emotion effects when the test face was last or third exited revealed that accuracy was significantly better for happy than angry faces when the test face was the last exited (β = 0.471, *SE* = 0.223, *z* = 2.11, *p* = .035), but not when it was the penultimate exited (β = −0.113, *SE* = 0.203, *z* = −0.56, *p* = .579), see Figure 6a. No other interactions between emotion and exit order were significant (maximum *z* = −0.706, minimum *p* = .480; Model 7.5).[Fig-anchor fig6]

##### Experiment 1: Precision

###### Continuous eye-movement predictors

Neither total fixation time (Model 8.1) nor mean fixation duration (Model 8.2) significantly predicted WM precision, regardless of the emotion conveyed at encoding (all *t*s < 1.60, all *p*s > .116). Regarding mean visit duration, while the main effect was not significant (β = 0.013, *SE* = 0.008, *t* = 1.72, *p* = .093), we found a significant interaction with emotion (β = 0.032, *SE* = 0.015, *t* = 2.23, *p* = .033; Model 8.3) indicating that longer mean visit durations benefited precision for happy faces more than for angry faces.

###### Selection order

No effects involving selection order were significant (maximum *t* = 0.66, minimum *p* = .509), indicating that precision of relocation did not depend on whether the test face during encoding was selected as first, second, third, or fourth, and this was independent of the emotional expression (Model 8.4).

###### Exit order

Precision was significantly enhanced when the test face was exited last compared with when it was exited third (β = 0.050, *SE* = 0.019, *t* = 2.64, *p* = .011), second (β = 0.056, *SE* = 0.020, *t* = 2.78, *p* = .008), or first (β = 0.040, *SE* = 0.018, *t* = 2.30, *p* = .023; Figure 6b). Facial emotion at encoding did not significantly modulate the impact of exit order on precision (maximum *t* = 1.33, minimum *p* = .065), although Figure 6b shows that a happy advantage is only observable at the last fixation exit (Model 8.5).

##### Experiment 2: Accuracy

###### Continuous eye-movement predictors

We did not find any significant effects of total fixation time (Models 9.1.1–9.1.3) or mean fixation duration (Models 9.2.1–9.2.3) on WM accuracy at any maintenance interval duration and regardless of emotion (all *t*s < 1.43, all *p*s > .152). We did find that longer mean visit duration on the test face significantly predicted greater WM accuracy at 3 s maintenance interval (β = 0.117, *SE* = 0.051, *t* = 2.31, *p* = .021; Model 9.3.2), but similarly for angry and happy faces (two-way interaction: β = −0.002, *SE* = 0.095, *t* = −0.02, *p* = .982). However, mean visit duration did not significantly predict accuracy at either 1 s or 6 s, regardless of emotion (all *t*s < 0.75, all *p*s > .940; Models 9.3.1 and 9.3.3). It is difficult to interpret why such an effect would be found only with the 3 s interval.

###### Selection order

**Figure 7 fig7:**
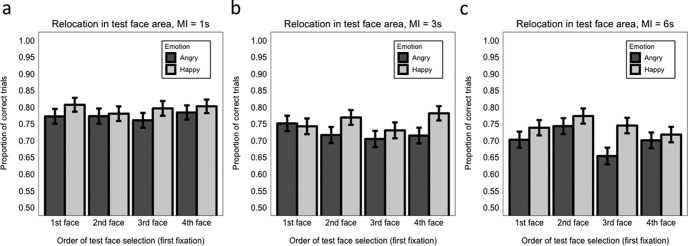
Accuracy in Experiment 2 as a function of fixation selection order for (a) 1-s, (b) 3-s, and (c) 6-s maintenance intervals (MIs).

As in Experiment 1, at 1 s maintenance interval no effects involving selection order were significant (maximum *z* = 0.81, minimum *p* = .418; Model 9.4.1), indicating that accuracy of relocation did not depend on whether the test face during encoding was selected first, second, third, or fourth, and this was regardless of the emotional expression conveyed (Figure 7a).[Fig-anchor fig7]

With 3 s maintenance interval, the interaction between emotion and first versus fourth (last) face selected was significant (β = 0.753, *SE* = 0.297, *z* = 2.53, *p* = .011; Model 9.4.2). Follow-up analyses examining separately the effect of emotion when the test face was first or last selected, revealed that the happy advantage on relocation accuracy was present only when the face was the last selected (happy face: *M* = 0.78, *SE* = 0.41 vs. angry face: *M* = 0.72, *SE* = 0.45; β = 0.554, *SE* = 0.224, *z* = 2.47, *p* = .014) but not when it was the first selected (β = −0.108, *SE* = 0.197, *z* = 0.55, *p* = .581; Models 9.4.2.1 and 9.4.2.2; Figure 7b). No other effects were significant (maximum *z* = 1.59, minimum *p* = .112).

With 6 s maintenance interval (Figure 7c), relocation accuracy was significantly better when the face to be tested was the second selected (*M* = 0.76, *SE* = 0.428) compared with when it was the first selected (*M* = 0.73, *SE* = 0.449; β = 0.345, *SE* = 0.159, *z* = 1.98, *p* = .048; Model 9.4.3). No other effects were significant (maximum *z* = 0.89, minimum *p* = .374).

###### Exit order

**Figure 8 fig8:**
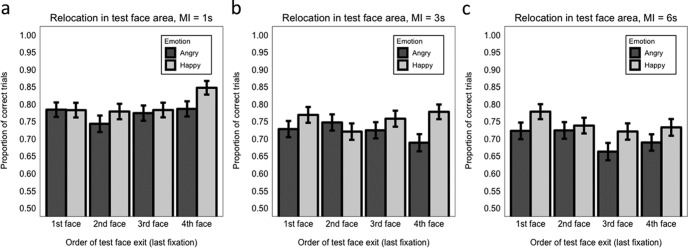
Accuracy in Experiment 2 as a function of exit fixation order for (a) 1-s, (b) 3-s, and (c) 6-s maintenance intervals (MIs).

With 1 s maintenance interval, we found that accuracy when the face to be tested was the last (fourth) exited was better than when it was exited in any other order (Figure 8a): last versus third (β = 0.293, *SE* = 0.147, *z* = 1.99 *p* = .029); last versus second (β = 0.465, *SE* = 0.148, *z* = 3.13, *p* = .002); last versus first (β = 0.340, *SE* = 0.147, *z* = 2.31, *p* = .021; Model 9.5.1). No interaction with emotion was significant (maximum *z* = 1.59, minimum *p* = .111). The pattern of results is largely comparable with what we found for Experiment 1 at load 4, with the exception that the last versus third (penultimate) exit comparison was not modulated by emotion here.[Fig-anchor fig8]

With 3 s maintenance interval (Figure 8b), the interaction between emotion and face exit last versus second was significant (β = 0.717, *SE* = 0.276, *z* = 2.60, *p* = .009; Model 9.5.2). Follow-up analysis, considering separately the effect of emotion when the test face was second or last exited, showed that a happy expression was beneficial for relocation accuracy when the face was last exited (β = 0.471, *SE* = 0.223, *z* = 2.11, *p* = .035), but there was no significant difference between happy and angry face accuracy when the face was the second exited (β = −0.128, *SE* = 0.194, *z* = −0.66, *p* = .508; Models 9.5.2.1 and 9.5.2.2). No other effects were significant (maximum *z* = 0.943, minimum *p* = .346).

With 6 s maintenance interval (Figure 8c), there were no significant effects (maximum *z* = 1.13, minimum *p* = .257; Model 9.5.3), indicating that relocation performance did not differ when the test face was the last exited compared with when it was the third, second, or first exited, and this was regardless of the emotional expression conveyed at encoding.

##### Experiment 2: Precision

###### Continuous eye-movement predictors

WM precision was not significantly affected by any of the inspection duration measures we considered (total fixation time, mean fixation duration and mean visit duration on the test face) or by the interaction between one of these predictors and emotion, and this was found across all maintenance intervals (maximum *t* = 1.90, minimum *p* = .058; Models 10.1.1–10.3.3).

###### Selection order

No effect involving selection order was significant at any maintenance interval (maximum *t* = 1.31, minimum *p* = .192; Model 10.4.1–10.4.3).

###### Exit order

**Figure 9 fig9:**
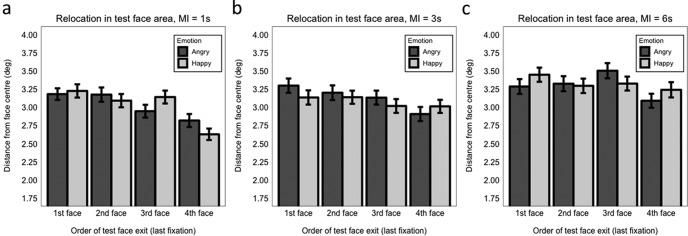
Precision in Experiment 2 as a function of exit fixation order for (a) 1-s, (b) 3-s, and (c) 6-s maintenance intervals (MIs).

With 1-s maintenance interval (Figure 9a), relocation precision was significantly higher when the test face was the last exited compared with when it was the third exited (β = 0.057, *SE* = 0.015, *t* = 3.69, *p* < .001), the second exited (β = 0.067, *SE* = 0.016, *t* = 4.38, *p* < .001), or the first exited (β = 0.057, *SE* = 0.015, *t* = 3.69, *p* < .001 (Model 10.5.1). No other effects were significant (maximum *t* = 0.36, minimum *p* = .096). Overall, this pattern of results reflects what we found at load 4 for Experiment 1.[Fig-anchor fig9]

With 3-s maintenance interval (Figure 9b), precision was enhanced when the test face was the last exited compared with both when it was the second exited (β = 0.037, *SE* = 0.018, *t* = 2.08, *p* = .044), or the first exited (β = 0.043, *SE* = 0.017, *t* = 2.47, *p* = .016; Model 10.5.2). Precision did not differ for test faces that were exited last versus third (penultimate: β = 0.022, *SE* = 0.017, *t* = 1.29, *p* = .202). No other effects were significant (maximum *t* = 1.90, minimum *p* = .059).

With 6-s maintenance interval (Figure 9c), precision was significantly improved only when the test face was the last exited compared with when it was the third exited (β = 0.042, *SE* = 0.017, *t* = 2.47, *p* = .014; Model 10.5.3). No other effects were significant (maximum *t* = 1.54, minimum *p* = .125).

### Results Summary: Oculomotor Activity

First, we considered whether the emotion conveyed by the faces at encoding modulated oculomotor inspection behavior (using only load 4 data in Experiment 1, comparable to Experiment 2 in which four faces were always displayed). In Experiment 1, there was no significant effect of emotion on mean fixation duration or mean visit duration per face. In Experiment 2, mean fixation duration per face was significantly longer for angry than happy faces, but this difference only amounted to 4 ms and was not found to relate to subsequent WM accuracy. No effect of emotion on mean visit duration was found in Experiment 2.

Second, we considered whether three continuous eye movement measures (total fixation time, mean fixation duration, and mean visit duration) predicted WM accuracy and precision. *Accuracy.* In Experiment 1, none of these oculomotor measures showed an effect on WM, or modulated WM differences between happy and angry faces. In Experiment 2, only longer mean visit duration with a 3 s maintenance interval significantly predicted better WM accuracy, but this did not influence the effect of emotion on WM identity-location binding. *Precision.* In Experiment 1, only mean visit duration modulated WM precision and only by interaction with emotion, showing that longer mean visit duration enhanced relocation precision for happy faces more than angry faces. However, the lack of a significant difference in precision performance between happy and angry faces means that we cannot draw any clear conclusions from this. In Experiment 2, no continuous oculomotor predictors influenced precision regardless of emotion and maintenance interval.

Third, we assessed whether the order in which the test face was selected for first fixation during encoding influenced WM accuracy and precision. *Accuracy.* Only in Experiment 2 did we find an influence of selection order but only with a 3 s or 6 s maintenance interval. With 3 s maintenance, there was a significant happy versus angry advantage in WM accuracy only when the test face happened to have been the last face selected. With 6 s maintenance, there was no modulation of selection order on emotion effects, but overall WM accuracy was better when the test face happened to have been selected second versus first. *Precision.* Across both experiments there were no significant effects of selection order on WM precision, regardless of emotion or maintenance interval.

Finally, we assessed whether the order in which the test face was exited (i.e., when it was finally fixated at encoding) influenced WM accuracy and precision. *Accuracy.* In Experiment 1, overall accuracy was significantly improved when the test face was last (fourth) exited (compared with when it was exited first or second) indicating a form of recency effect in identity-location binding. Further, when the last versus penultimate exit orders were compared, a significant happy versus angry advantage was only found when the test face had been exited last. In Experiment 2, using a 1-s maintenance interval (the same maintenance interval used in Experiment 1), there was better accuracy overall when the test face was last exited versus when it was the first, second, or third face exited, largely replicating the general recency effect found in Experiment 1. The heightened happy advantage for last versus penultimate exit was not replicated at 1 s but was replicated at 3 s. There were no effects of exit order at 6-s maintenance interval regardless of emotion. Thus, broadly speaking, there appears to be a recency effect wherein happy faces were relocated more accurately than angry faces mainly when the test face was exited last, but this effect was only observed when the maintenance interval was relatively short (1 s or 3 s). *Precision.* In both experiments (and across all maintenance intervals in Experiment 2) in general precision was significantly better when the test face was exited last, but this recency effect was not modulated by emotion.

## Discussion

Here we reported two experiments in which participants were required to encode both the identity and location of a number of angry or happy faces into visuospatial WM. Emotional expression was irrelevant to the task. After a short blank maintenance interval, one centrally presented and neutrally expressive test face appeared and always matched identity to one of the faces just seen for encoding. Participants used their index finger on the touchscreen to relocate this face to its original position, as precisely as they could, with no placeholders to aid relocation. Our paradigm improves on several shortfalls of the face relocation task used by Terburg et al. (2012), and additional measures and manipulations were applied for more in-depth investigation of how emotional expression influences face identity-location binding in WM. In both experiments, we found a happy face advantage that was not modulated by WM load or maintenance interval (1 s to 6 s), social anxiety, autistic-like traits, or current mood, indicating a robust prosocial effect of emotional expression on remembering who was where. Few of the oculomotor measures during encoding contributed to WM performance or to the WM advantage of happy compared with angry faces. However, a novel finding was uncovered where the happy benefit was most evident when the to-be-tested face happened to have been the last face fixated and exited at encoding, immediately prior to the maintenance phase.

First we discuss in more detail the specific influence of emotion on identity-location binding, then the oculomotor results. We also discuss the more general effects of load and maintenance interval on WM performance.

### Effect of Emotional Expression on WM Performance

In both experiments, we measured WM relocation performance in two ways: (1) broad “accuracy” and (2) finer-grained “precision.” Accuracy was computed as the proportion of trials on which the test face was relocated within 7 degrees of the center of the original face location (coded “correct” within this zone and “incorrect” outside this zone). Precision was computed as the distance between the center of the correctly relocated test face and the original face location within the 7-degree zone. While relocation accuracy was significantly higher when faces showed a happy versus angry expression at encoding, precision was unaffected by emotional expression (this was also found when precision computation was unconstrained by the 7-degree zone). This indicates that only coarse-grained identity-location binding was influenced by emotion. Being able to determine that the presence of a happy expression benefits only coarse identity-location bindings and not more fine-grained precision is important, as it suggests that good approximations of person location are sufficient. Previous research has stated that there are two mechanisms by which object-location binding can be achieved, one according to exact position, known as “coordinate binding”, and another according to a less precise approximation of location, known as “categorical binding” (e.g., Kosslyn, Chabris, Marsolek, & Koenig, 1992; van Asselen, Kessels, Kappelle, & Postma, 2008). Furthermore, categorical binding is thought to be engaged during object-location memory tasks using multiple objects and locations (Alexander, Packard, & Peterson, 2002), where object locations are encoded in relation to other objects in an allocentric manner (see Postma, Kessels, & van Asselen, 2008 for an overview). Thus, in the current study using social stimuli, categorical binding of who was where may also dominate over coordinate binding yielding the happy benefit in accuracy but not precision.

The happy advantage found here aligns with Terburg et al.’s (2012) findings and the happy (vs. fear and neutral) advantage in Gonzáles-Garrido et al.’s (2013) study. However, our study is the first to show specifically and directly that a happy expression at encoding enhances the binding between face identity and location in WM. Terburg and colleagues required participants to reposition multiple test faces according to both identity and expression (encoding displays contained a mix of angry/neutral or happy/neutral faces with repeated identity), thus there is no ability to isolate identity-location binding effects. Our paradigm was able to do this by making expression task irrelevant, and presenting encoding displays that were homogeneous with respect to expression and heterogeneous with respect to identity. In Gonzáles-Garrido and colleagues’ study, both expression and face identity information were task irrelevant and the task simply required participants to repeat the spatiotemporal sequence of face presentation at encoding, which thus requires no binding of any face information. We further add to existing literature by showing that the effect of emotional expression on face-identity-location bindings was unaffected by WM load or maintenance interval. In particular, lack of influence by maintenance interval length on the happy advantage suggests that, although happy face-location bindings appear to be more accurate, this does not then lead to superior maintenance of these bindings over time. Accuracy declined similarly for happy and angry faces as maintenance interval increased.

The happy benefit in visuospatial WM found here contrasts with the angry benefit found in tasks that examine only visual WM for face identity in the absence of location information (e.g., Jackson et al., 2008, 2009, 2014). This may be surprising, considering the similarities in encoding and retrieval demands we took care to employ here (with reference to Jackson et al., 2014: multiple faces with the same emotional expression at encoding and a single neutrally expressive test face at retrieval). Yet both the angry visual WM benefit and the happy visuospatial WM benefit appear to be robust and replicate across experiments. So why do we not find a threat advantage here, why is the ability to relocate a specific face using WM enhanced when that face was smiling?

It is clear that each task engages different perceptual and mnemonic processes, both visual (identity) and spatial (location) processes here, but only visual (identity) processes in prior studies (e.g., Jackson et al., 2014). It is known that the hippocampus (HPC) is associated with spatial WM (see Logie, 2014), and complex high-resolution perceptual or conceptual binding (Yonelinas, 2013) and binding (swap) errors (Liang et al., 2016; Pertzov et al., 2013). In addition, the HPC is recruited in the regulation of approach and avoid behaviors (see Ito & Lee, 2016). Therefore, while we have no direct evidence for this, it may be logical to assume that the HPC is recruited to a greater extent in the current visuospatial WM task than the purely visual WM task used in previous studies (Jackson et al., 2014). Indeed, Jackson et al. (2008) found that the angry versus happy benefit in visual WM was associated with increased activity on angry face trials in right lateralized inferior frontal sulcus, superior temporal sulcus, and globus pallidus, with no significant modulation of the HPC by emotional expression found in the whole-brain analyses. If HPC was recruited during the current visuospatial task, it is therefore possible that approach/avoid mechanisms were also engaged to a greater extent. Different emotions expressed by another individual can signal an *intention* to either approach or avoid: both happy and angry expressions are thought to signal approach (benevolent or malevolent, respectively), while fear signals avoidance of something or someone (Adams & Kleck, 2003, 2005). Signals of intent conveyed by another also elicit an approach or avoid *response* in the observer: here, the evidence shows that angry and happy expressions diverge, with happy faces found to elicit an approach response, while angry faces elicit an avoid response (Marsh, Ambady, & Kleck, 2005; Seidel, Habel, Kirschner, Gur, & Derntl, 2010). Happy faces, therefore, are congruent with respect to an approach signal and approach response, while angry faces are incongruent with an approach signal but an avoid response (Adams, Ambady, Macrae, & Kleck, 2006). We speculate that perhaps this congruency of signal and response for happy faces led to better location-identity binding.

The effect of signal/response congruency may be further influenced by the nature of the retrieval response used here: participants were forced to execute a motoric “approach” response by moving their hand and arm toward the test face, and touch it to move it to its original location. Thus, for angry faces not only is there conflict between signal (approach) and natural response tendency (avoid), but potentially also between response tendency (avoid) and response execution (approach). Such response tendency/execution conflict has been demonstrated by Bamford and Ward (2008). Using a speeded forced-choice selection task on a touchscreen computer, they showed that participants were faster to approach (touch) pleasant words or pictures and slower to approach unpleasant stimuli (Bamford & Ward, 2008; Experiment 1). Our WM task did not require a speeded response, there was no response selection choice, and on each trial participants could position then reposition the test face until they were content with its location. Thus, there is no rationale or logic to examine RTs in our study. However, further research using a different relocation method that does not involve the physical approach response used here (such as using a mouse, keyboard keys, or eye movements to relocate the test face) may help determine whether response tendency/execution conflict during the retrieval phase does play a role or not in how emotion influences visuospatial WM for who was where.

An additional explanation for the happy advantage found here may be that happy faces were processed with greater fluency than angry faces (Becker & Srinivasan, 2014), and this may have released more resources for visuospatial processing in WM. In support of this, in Experiment 2 we found that mean fixation duration per face was slightly shorter (only by 4 ms on average) when faces were happy versus angry, which might indicate more efficient information gathering. However, it must be noted that in contrast during a purely visual WM task memory was enhanced for face identity information when faces were angry versus happy (e.g., Jackson et al., 2008, 2009, 2014). Perhaps processing and resource efficiencies in WM may change between different task contexts and demands. When both identity and location are to be encoded into WM, the approach/avoid mechanisms that we propose to be engaged here may drive the degree of fluency. Specifically, greater resource efficiency may be achieved when there is congruency between an approach signal and an approach response, as we have here with happy faces, compared with when there is signal-response conflict (as for angry faces in this spatial relocation task: approach signal, avoid response).

It could alternatively be claimed that smiling faces with exposed teeth are more perceptually salient than angry faces and, therefore, boost WM for who was where simply via low-level, non-social mechanisms (all of the happy faces used here had teeth showing, while none of the angry faces showed teeth; e.g., Becker et al., 2011; Horstmann, Lipp, & Becker, 2012). Becker et al. (2011) suggested that exposed teeth seen in the established form of a happy expression may be designed to be detectable by evolution and hence may have adaptive functions. The sensory bias hypothesis argues, indeed, that selection pressures shaped facial signals to suit the capabilities of the human visual system and, as a result, the most vital signals became perceptually prominent (Horstmann & Bauland, 2006). However, this does not explain why a happy advantage is not similarly found when just visual WM for face identity is tested (Jackson et al., 2008, 2009, 2014). In those studies, all happy faces showed teeth while 50% of angry faces showed teeth, and in Jackson et al. (2008, 2009) there was no difference in visual WM for happy versus neutral faces (all neutral faces had mouth closed). Unlike visual search tasks (e.g., Becker et al., 2011; Horstmann et al., 2012), which measure the speed to detect the presence or absence of a singularly different emotion in a crowd (e.g., one happy face or one angry face in a crowd of neutral faces, or vice versa), in the current study encoding displays were homogeneous with regard to expression (all happy or all angry), so there is no opportunity for a smiling face to perceptually stand out somehow from the others because of saliency of expressive features. Furthermore, other visual WM research has shown that when a heterogeneous encoding display was used in which there was a singleton happy face among three neutral faces, recall accuracy of face identity was no different for happy versus neutral faces. However, visual WM was significantly better for an angry singleton compared with the concurrently presented neutral faces (Thomas, Jackson, & Raymond, 2014). Thus, it seems unlikely that low-level featural artifacts could fully account for the results found here, and a prosocial navigation account is more plausible.

We now turn to the role of eye movements during encoding. We assessed (1) whether there were any differences in oculomotor behavior during encoding of happy versus angry faces, and (2) whether oculomotor behavior at encoding could predict WM accuracy and precision.

### Oculomotor Behavior

First, we were interested in whether oculomotor inspection behavior was different when participants viewed angry versus happy faces at encoding. Measures of mean fixation duration and mean visit duration per face showed no effect of emotion in Experiment 1. In Experiment 2 participants fixated each angry face on average for significantly longer than they fixated each happy face, but note that this difference amounted to only 4 ms, and clearly did not serve to enhance visuospatial WM for angry faces. We conducted further analyses to determine whether total fixation time, mean fixation duration, and mean visit duration on the face-to-be-tested at encoding (unbeknownst to participants) could explain the happy advantage. The time spent looking specifically at the to-be-tested face during encoding, regardless of how this was sampled, was not able to explain better WM for happy versus angry faces.

What did emerge, however, was an oculomotor recency effect in which a significant happy versus angry advantage was limited to trials in which the test face happened to have been the last face fixated at the end of the encoding period. This recency effect for the happy advantage was found only with accuracy and not precision measures of performance, and was also restricted to short maintenance intervals (1 s or 3 s), indicating a short time window between perception and recall in which emotional expression exerts an effect. When we examined emotion effects according to fixation selection order (the temporal position in which the test face was first fixated at encoding: first, second, third, or fourth), we found a significant happy versus angry advantage (on accuracy data only) when the test face happened to have been the last face selected, but only with a 3 s maintenance interval. This again indicates that the enhancement effect in WM for happy faces is time-sensitive—it is observed when the time between perception and recall is minimized and abolished when a longer amount of time has elapsed. It is clear that early examination of the test face at encoding is not beneficial. These fixation order effects also suggest that the happy advantage may be most robust when there are few or no intervening fixations on other faces during encoding before a relocation response is required, and is thus sensitive to potential interference effects.

We now discuss the more general effects of load and maintenance interval on WM performance that were found.

### General Effects of Load on Accuracy and Precision

In Experiment 1, we presented one, two, three, or four faces for encoding. Both accuracy and precision declined as load increased. For the accuracy data, we observed a linear decline from Load 2 to 4. This decline in accuracy could reflect weaker identity-location bindings as a result of increased encoding demands, or alternatively result simply from the fact that as load increased there were more locations in which to erroneously reposition the test face. To clarify this, we considered the effect of load on swap errors, where participants placed the test face within 7-degree of a different face and a mis-binding of who was where is thought to have occurred. If reduced accuracy was simply a product of greater chance for swap errors as the number of possible locations to relocate the test face increased, then the proportion of swap errors to random errors is predicted to have increased as a function of load. However, in Experiment 1 we found that the proportion of swap errors was not significantly affected by load. Errors at all loads were predominantly because of mis-binding rather than random relocation (92.64% on average), indicating that participants had a clear WM representation for all face locations regardless of load, and suggesting that what was affected by load was specifically the binding of identity to those locations.

The precision results show that, on trials in which they knew the correct location of the test face, participants were reliably precise in relocating the face in the 7-degree “correct” region. Even with four faces to encode, precision was on average 2.59 degrees of visual angle in Experiment 1 (3.03 degrees in Experiment 2 with the same 1-s maintenance interval), which equates to approximately 1.8 to 2.1 cm of error. It is interesting to note that precision was poorer for test faces incorrectly placed in the location of another, co-occurring face (swap errors) compared with correctly relocated test faces. This suggests some qualitative difference in the nature of a correctly bound identity-location representation in WM, versus a broadly accurate spatial representation that has become disconnected from its original visual content.

Precision declined as load increased, but rather than a linear decline we observed a plateau effect in which precision declined significantly from Load 2 to 3 but not from Load 3 to 4. This may reflect a threshold of representational fidelity that is reached when discrete item-limits in WM are reached, in accordance with a hybrid account of WM capacity, which combines a discrete-resource “slots” model with flexible resource models (Zhang & Luck, 2008; see Ma, Husain, & Bays, 2014 for an overview of WM capacity models). Purer flexible- or shared-resource models, on the other hand, would predict a continual decline in precision as load increases (e.g., Bays, 2015; Bays et al., 2009), reflecting a graded reduction in amount of resources that can be allocated to each item as the number of items increases. Our asymptote could simply be an artifact of the inclusion of fewer correct trials at Load 4 than 3 in the 7-degree constrained analysis. However, even when we computed precision without the constraint of the 7-degree correct region, precision significantly declined from two to three faces but not from three to four faces, indicating that this is not the case.

The pattern of our precision data do not converge with those of Pertzov et al. (2012), who found a linear decline in object-location binding precision from one to five objects. This difference between our study and Pertzov et al.’s pattern of results could stem from differences in WM capacity for objects versus faces. Pertzov and colleagues used simple objects that could have been easily discriminated on the basis of shape, outline, and color, while we used greyscale faces, which would have been much harder to discriminate on these properties—we wanted to force participants to encode internal facial information rather than external features, to assess face identity memory specifically. A study by Lorenc, Pratte, Angeloni, and Tong (2014) investigated WM precision for faces by using a continuous face space in which different faces were morphed into one another and used as the retrieval test stimulus. Participants were shown one, three, or five faces presented sequentially at different locations on the screen for encoding, and after a 3 s maintenance interval a cue indicated the location of the face to report from memory. Participants then cycled through 80 possible face morphs from the continuum to select the closest match as a measure of precision. They found that overall precision declined significantly as load increased, and specific contrasts showed a significant decline between one face and five faces, but not between three and five faces. Thus, Lorenc et al.’s findings indicate a form of asymptote in precision for face WM identification similar to the one found here for more specific face identity-location binding. However, we did not set out to explicitly test discrete- versus flexible-resource models in the current study, so we do not make any explicit conclusions from our findings. Further work is required to explore this appropriately.

### Effect of Maintenance Interval on Accuracy and Precision

In Experiment 2, we used always four faces at encoding and manipulated the maintenance interval to a duration of 1 s, 3 s, or 6 s. Accuracy declined in a linear fashion from 1 s to 3 s and from 3 s to 6 s. Precision within the 7-degree zone of the correct face location did not decline from 1 s to 3 s, but did significantly decrease from 3 s to 6 s, and without the constraint of the 7-degree zone precision also declined significantly between 1 s and 3 s. Thus, both coarse- and fine-grained binding of identity-location information weakened over time. Furthermore, the number of swap errors in proportion to random errors significantly decreased from 3 s to 6 s, indicating that participants were more likely to make random errors at the longest delay. This increase in random errors at 6 s suggests that spatial WM for the face locations alone also declines. Reduced accuracy and precision for bound information with increased maintenance interval is in line with findings from Pertzov et al. (2012). Pertzov, Manohar, and Husain (2017) propose that reduced precision over time is not just because of a temporal decay of the bound representation, but also because of interference between stored items, which can degrade or weaken the bindings.

Finally, it could be argued that the effects we see here may have been driven not by binding failures per se, but purely by identity failures with locational WM remaining intact (see Pertzov et al., 2012). To mitigate this, Pertzov and colleagues amended the object-location paradigm to include two objects at test, only one of which was there at encoding (Liang et al., 2016; Pertzov et al., 2012, Experiments 2 and 3; Pertzov et al., 2013; Pertzov, Heider, Liang, & Husain, 2015). Participants had to select the object they thought was there before relocating it, and data were analyzed only on trials in which the correct object was selected. Thus, there was higher confidence that identity memory was intact. Here we used a single test item as it is currently unknown at present how this additional identity-probe task may interfere with bound representations in visuospatial WM. It is reasonable to question this because in a different task context it has been shown that new perceptual input can interfere with the contents of WM (e.g., Allen, Baddeley, & Hitch, 2006; Alvarez & Thompson, 2009). This requires further careful examination in visuospatial WM, especially in the context of faces and the specific task design, which we adopted here. In our task, to encourage face identity abstraction over template matching the faces were emotional at encoding and neutral at test (as per Jackson et al., 2014). This also engages a form of WM manipulation across encoding and retrieval phases, in addition to storage. Visuospatial WM for faces in this task may therefore be more resource demanding than the image-based matching of non-manipulated objects or fractals employed by Pertzov and colleagues, and potentially be more vulnerable to interference at recall by an additional face identification task. Perceptual similarity effects between the encoding faces and the foil face would also have to be considered, as increased similarity between items has been shown to decrease visual recall accuracy (e.g., Jackson, Linden, Roberts, Kriegeskorte, & Haenschel, 2015; Wood, 2011). These are key points to consider for future research. As an alternative, a single test face could be presented, which was either present or absent at encoding, and participants only relocate the face if they thought it was present. However, the proportion of present to absent trials would need to be 50/50 to avoid response biases, and this would mean that half (or more) of the trials would be excluded from analysis and make the whole task unwieldy.

To address the question of whether our results reflect purely face identity errors rather than binding impairments, we can examine our current data. As mentioned previously, two item-location binding mechanisms have been distinguished—categorical binding and coordinate binding (Kosslyn et al., 1992; Van Asselen et al., 2008). The contribution of categorical spatial WM errors to performance can be considered by examining swap errors, while coordinate spatial WM errors are reflected in our precision measure. The high proportion of swap to random errors in Experiment 1 here could be taken to indicate little or no loss of categorical spatial WM information, because more random relocation errors would be expected if categorical locations were poorly recalled. In Experiment 1, one could predict that increasing load should impair both locational and identity WM, and yield a decline in the proportion of swap errors. However, Figure 3a shows that swap errors in fact became more common as load increased, although this was nonsignificant (perhaps because of near ceiling values and the low number of error data points at lower loads). It is important to note that predicting how load may impact swap errors is tricky, because it is confounded by increased opportunity to make more swap errors as the number of locations to swap with increases. Our additional measure of relocation precision does show a significant decline as load increases (Figure 2b). This indicates that, on trials in which the test face was correctly relocated within 7 degrees from the center of its original location, finer-grained coordinate spatial WM was impaired as the number of locations increased. In addition, precision within the 7-degree zone was poorer on swap error trials than on correct trials, suggesting that both identity and coordinate locational information was degraded here (although this was only a tendency toward significance in Experiment 1, it was significant in Experiment 2). In Experiment 2, the proportion of swap errors and precision on correct trials decreased as maintenance interval increased, indicating that both categorical and coordinate spatial information was degraded. Thus, our data indicate that face identity was not solely impaired and our results are interpreted to reflect binding mechanisms.

## Conclusion

In conclusion, the binding between face identity and location in WM is more accurate when faces are encoded with a happy expression than an angry expression. Becker and colleagues posit that a smiling face conveys a number of social signals that were vital for our ancestors’ survival, such as acceptance and cooperation (Becker et al., 2011). This prosocial effect could be an adaptive approach response, which enhances social networking and person navigation, and indicates a desire to connect and reconnect with individuals who may prove beneficial to our future social and emotional welfare.

## Supplementary Material

10.1037/xlm0000522.supp

## References

[c1] Adams R. B. Jr., Ambady N., Macrae C. N., & Kleck R. E. (2006). Emotional expressions forecast approach-avoidance behavior. Motivation and Emotion, 30, 177–188. 10.1007/s11031-006-9020-2

[c2] Adams R. B. Jr., & Kleck R. E. (2003). Perceived gaze direction and the processing of facial displays of emotion. Psychological Science, 14, 644–647. 10.1046/j.0956-7976.2003.psci_1479.x 14629700

[c3] Adams R. B. Jr., & Kleck R. E. (2005). Effects of direct and averted gaze on the perception of facially communicated emotion. Emotion, 5, 3–11. 10.1037/1528-3542.5.1.3 15755215

[c4] Alexander G. M., Packard M. G., & Peterson B. S. (2002). Sex and spatial position effects on object location memory following intentional learning of object identities. Neuropsychologia, 40, 1516–1522. 10.1016/S0028-3932(01)00215-9 11931956

[c101] Allen R. J., Baddeley A. D., & Hitch G. J. (2006). Is the binding of visual features in working memory resource-demanding? Journal of Experimental Psychology: General, 135, 298–313. 16719655 10.1037/0096-3445.135.2.298

[c102] Alvarez G. A., & Thompson T. W. (2009). Overwriting and rebinding: Why feature-switch detection tasks underestimate the binding capacity of visual working memory. Visual Cognition, 17, 141–159.

[c5] Bamford S., & Ward R. (2008). Predispositions to approach and avoid are contextually sensitive and goal dependent. Emotion, 8, 174–183. 10.1037/1528-3542.8.2.174 18410191

[c6] Bannerman R. L., Temminck E. V., & Sahraie A. (2012). Emotional stimuli capture spatial attention but do not modulate spatial memory. Vision Research, 65, 12–20. 10.1016/j.visres.2012.05.011 22664375

[c7] Baron-Cohen S., Wheelwright S., Skinner R., Martin J., & Clubley E. (2001). The autism-spectrum quotient (AQ): Evidence from Asperger syndrome/high-functioning autism, males and females, scientists and mathematicians. Journal of Autism and Developmental Disorders, 31, 5–17. 10.1023/A:1005653411471 11439754

[c8] Barr D. J., Levy R., Scheepers C., & Tily H. J. (2013). Random effects structure for confirmatory hypothesis testing: Keep it maximal. Journal of Memory and Language, 68, 255–278. 10.1016/j.jml.2012.11.001 PMC3881361 24403724

[c104] Bates D., Maechler M., Bolker B., & Walker S. (2014). lme4: Linear mixed-effects models using Eigen and S4 (R Package Version 1.1–23). Retrieved from https://CRAN.R-project.org/package=lme4

[c9] Bays P. M. (2015). Spikes not slots: Noise in neural populations limits working memory. Trends in Cognitive Sciences, 19, 431–438. 10.1016/j.tics.2015.06.004 26160026

[c10] Bays P. M., Catalao R. F. G., & Husain M. (2009). The precision of visual working memory is set by allocation of a shared resource. Journal of Vision, 9, 7 10.1167/9.10.7 PMC3118422 19810788

[c11] Becker D. V., Anderson U. S., Mortensen C. R., Neufeld S. L., & Neel R. (2011). The face in the crowd effect unconfounded: Happy faces, not angry faces, are more efficiently detected in single- and multiple-target visual search tasks. Journal of Experimental Psychology: General, 140, 637–659. 10.1037/a0024060 21744984

[c12] Becker D. V., Mortensen C. R., Anderson U. S., & Sasaki T. (2014). Out of sight but not out of mind: Memory scanning is attuned to threatening faces. Evolutionary Psychology, 12, 901–912. 10.1177/147470491401200504 25350953 PMC10434408

[c13] Becker D. V., & Srinivasan N. (2014). The vividness of the happy face. Current Directions in Psychological Science, 23, 189–194. 10.1177/0963721414533702

[c14] Bradley B. P., Mogg K., Falla S. J., & Hamilton L. R. (1998). Attentional bias for threatening facial expressions in anxiety: Manipulation of stimulus duration. Cognition and Emotion, 12, 737–753. 10.1080/026999398379411

[c15] Feldmann-Wüstefeld T., Schmidt-Daffy M., & Schubö A. (2011). Neural evidence for the threat detection advantage: Differential attention allocation to angry and happy faces. Psychophysiology, 48, 697–707. 10.1111/j.1469-8986.2010.01130.x 20883506

[c16] Findlay J. M., & Gilchrist I. D. (2003). Active vision: The psychology of looking and seeing. New York, NY: Oxford University Press 10.1093/acprof:oso/9780198524793.001.0001

[c17] Fox E., & Damjanovic L. (2006). The eyes are sufficient to produce a threat superiority effect. Emotion, 6, 534–539. 10.1037/1528-3542.6.3.534 16938095 PMC1852642

[c18] Fox E., Russo R., Bowles R., & Dutton K. (2001). Do threatening stimuli draw or hold visual attention in subclinical anxiety? Journal of Experimental Psychology: General, 130, 681–700. 10.1037/0096-3445.130.4.681 11757875 PMC1924776

[c19] Fukuda K., Awh E., & Vogel E. K. (2010). Discrete capacity limits in visual working memory. Current Opinion in Neurobiology, 20, 177–182. 10.1016/j.conb.2010.03.005 20362427 PMC3019116

[c20] Gonzáles-Garrido A. A., Gómez-Velázquez F. R., Sequeira H., Ramos-Loyo J., & López-Franco A. L. (2013). Gender differences in visuospatial working memory: Does emotion matter? International Journal of Psychological Studies, 5, 11–21.

[c21] Horstmann G., & Bauland A. (2006). Search asymmetries with real faces: Testing the anger-superiority effect. Emotion, 6, 193–207. 10.1037/1528-3542.6.2.193 16768552

[c22] Horstmann G., Lipp O. V., & Becker S. I. (2012). Of toothy grins and angry snarls: Open mouth displays contribute to efficiency gains in search for emotional faces. Journal of Vision, 12, 7 10.1167/12.5.7 22637708

[c105] Ito R., & Lee A. C. H. (2016). The role of the hippocampus in approach-avoidance conflict decision-making: Evidence from rodent and human studies. Behavioural Brain Research, 313, 345–357. 27457133 10.1016/j.bbr.2016.07.039

[c23] Jackson M. C., Linden D. E. J., & Raymond J. E. (2014). Angry expressions strengthen the encoding and maintenance of face identity representations in visual working memory. Cognition and Emotion, 28, 278–297. 10.1080/02699931.2013.816655 23895082

[c24] Jackson M. C., Linden D. E. J., Roberts M. V., Kriegeskorte N., & Haenschel C. (2015). Similarity, not complexity, determines visual working memory performance. Journal of Experimental Psychology: Learning, Memory, and Cognition, 41, 1884–1892. 10.1037/xlm0000125 26010826

[c25] Jackson M. C., Wolf C., Johnston S. J., Raymond J. E., & Linden D. E. J. (2008). Neural correlates of enhanced visual short-term memory for angry faces: An FMRI study. PLoS ONE, 3, e3536 10.1371/journal.pone.0003536 18958158 PMC2568825

[c26] Jackson M. C., Wu C.-Y., Linden D. E. J., & Raymond J. E. (2009). Enhanced visual short-term memory for angry faces. Journal of Experimental Psychology: Human Perception and Performance, 35, 363–374. 10.1037/a0013895 19331494

[c27] Juth P., Lundqvist D., Karlsson A., & Ohman A. (2005). Looking for foes and friends: Perceptual and emotional factors when finding a face in the crowd. Emotion, 5, 379–395. 10.1037/1528-3542.5.4.379 16366743

[c28] Kliegl R., Masson M. E. J., & Richter E. M. (2010). A linear mixed model analysis of masked repetition priming. Visual Cognition, 18, 655–681. 10.1080/13506280902986058

[c29] Kosslyn S. M., Chabris C. F., Marsolek C. J., & Koenig O. (1992). Categorical versus coordinate spatial relations: Computational analyses and computer simulations. Journal of Experimental Psychology: Human Perception and Performance, 18, 562–577. 10.1037/0096-1523.18.2.562 1593235

[c30] Kuznetsova A., Bruun Brockhoff P., & Haubo Bojesen Christensen R. (2016). lmerTest: Tests in Linear Mixed Effects Models (R package version 2.0–32). Retrieved from https://CRAN.R-project.org/package=lmerTest

[c31] Langner O., Dotsch R., Bijlstra G., Wigboldus D. H. J., Hawk S. T., & van Knippenberg A. (2010). Presentation and validation of the Radboud faces database. Cognition and Emotion, 24, 1377–1388. 10.1080/02699930903485076

[c32] Levi D. M. (2008). Crowding: An essential bottleneck for object recognition: A mini-review. Vision Research, 48, 635–654. 10.1016/j.visres.2007.12.009 18226828 PMC2268888

[c33] Liang Y., Pertzov Y., Nicholas J. M., Henley S. M. D., Crutch S., Woodward F., . . . Husain M. (2016). Visual short-term memory binding deficit in familial Alzheimer’s disease. Cortex: A Journal Devoted to the Study of the Nervous System and Behavior, 78, 150–164. 10.1016/j.cortex.2016.01.015 27085491 PMC4865502

[c34] Logie R. H. (2014). Visuo-spatial working memory. Hove, UK: Psychology Press.

[c35] Lorenc E. S., Pratte M. S., Angeloni C. F., & Tong F. (2014). Expertise for upright faces improves the precision but not the capacity of visual working memory. Attention, Perception, & Psychophysics, 76, 1975–1984. 10.3758/s13414-014-0653-z PMC4163543 24627213

[c36] Ma W. J., Husain M., & Bays P. M. (2014). Changing concepts of working memory. Nature Neuroscience, 17, 347–356. 10.1038/nn.3655 24569831 PMC4159388

[c37] Marsh A. A., Ambady N., & Kleck R. E. (2005). The effects of fear and anger facial expressions on approach- and avoidance-related behaviors. Emotion, 5, 119–124. 10.1037/1528-3542.5.1.119 15755225

[c38] Mennin D. S., Fresco D. M., Heimberg R. G., Schneier F. R., Davies S. O., & Liebowitz M. R. (2002). Screening for social anxiety disorder in the clinical setting: Using the Liebowitz Social Anxiety Scale. Journal of Anxiety Disorders, 16, 661–673. 10.1016/S0887-6185(02)00134-2 12405524

[c39] Mogg K., Millar N., & Bradley B. P. (2000). Biases in eye movements to threatening facial expressions in generalized anxiety disorder and depressive disorder. Journal of Abnormal Psychology, 109, 695–704. 10.1037/0021-843X.109.4.695 11195993

[c106] Oldfield R. C. (1971). The assessment and analysis of handedness: The Edinburgh inventory. Neuropsychologia, 9, 97–113. 5146491 10.1016/0028-3932(71)90067-4

[c40] Pertzov Y., Avidan G., & Zohary E. (2009). Accumulation of visual information across multiple fixations. Journal of Vision, 9, 2 10.1167/9.10.2 19810783

[c41] Pertzov Y., Dong M. Y., Peich M.-C., & Husain M. (2012). Forgetting what was where: The fragility of object-location binding. PLoS ONE, 7, e48214 10.1371/journal.pone.0048214 23118956 PMC3485137

[c42] Pertzov Y., Heider M., Liang Y., & Husain M. (2015). Effects of healthy ageing on precision and binding of object location in visual short-term memory. Psychology and Aging, 30, 26–35. 10.1037/a0038396 25528066 PMC4360752

[c43] Pertzov Y., Manohar S., & Husain M. (2017). Rapid forgetting results from competition over time between items in visual working memory. Journal of Experimental Psychology: Learning, Memory, and Cognition, 43, 528–536. 10.1037/xlm0000328 PMC5377990 27668485

[c44] Pertzov Y., Miller T. D., Gorgoraptis N., Caine D., Schott J. M., Butler C., & Husain M. (2013). Binding deficits in memory following medial temporal lobe damage in patients with voltage-gated potassium channel complex antibody-associated limbic encephalitis. Brain: A Journal of Neurology, 136, 2474–2485. 10.1093/brain/awt129 23757763 PMC3722347

[c45] Pinkham A. E., Griffin M., Baron R., Sasson N. J., & Gur R. C. (2010). The face in the crowd effect: Anger superiority when using real faces and multiple identities. Emotion, 10, 141–146. 10.1037/a0017387 20141311

[c46] Pool E., Brosch T., Delplanque S., & Sander D. (2016). Attentional bias for positive emotional stimuli: A meta-analytic investigation. Psychological Bulletin, 142, 79–106. 10.1037/bul0000026 26390266

[c47] Postma A., Kessels R. P., & van Asselen M. (2008). How the brain remembers and forgets where things are: The neurocognition of object-location memory. Neuroscience and Biobehavioral Reviews, 32, 1339–1345. 10.1016/j.neubiorev.2008.05.001 18562002

[c107] Proctor R. W., & Reeve T. G. (Eds.). (1989). Stimulus-response compatibility: An integrated perspective (Vol. 65). North Holland, the Netherlands: Elsevier.

[c48] Seidel E. M., Habel U., Kirschner M., Gur R. C., & Derntl B. (2010). The impact of facial emotional expressions on behavioral tendencies in women and men. Journal of Experimental Psychology: Human Perception and Performance, 36, 500–507. 10.1037/a0018169 20364933 PMC2852199

[c49] Sessa P., Luria R., Gotler A., Jolicœur P., & Dell’acqua R. (2011). Interhemispheric ERP asymmetries over inferior parietal cortex reveal differential visual working memory maintenance for fearful versus neutral facial identities. Psychophysiology, 48, 187–197. 10.1111/j.1469-8986.2010.01046.x 20557488

[c50] Stiernströmer E. S., Wolgast M., & Johansson M. (2016). Effects of facial expression on working memory. International Journal of Psychology, 51, 312–317. 10.1002/ijop.12194 26238683

[c51] Terburg D., Aarts H., & van Honk J. (2012). Memory and attention for social threat: Anxious hypercoding-avoidance and submissive gaze aversion. Emotion, 12, 666–672. 10.1037/a0027201 22309731

[c52] Thomas P. M. J., Jackson M. C., & Raymond J. E. (2014). A threatening face in the crowd: Effects of emotional singletons on visual working memory. Journal of Experimental Psychology: Human Perception and Performance, 40, 253–263. 10.1037/a0033970 23957307

[c53] van Asselen M., Kessels R. P. C., Kappelle L. J., & Postma A. (2008). Categorical and coordinate spatial representations within object-location memory. Cortex: A Journal Devoted to the Study of the Nervous System and Behavior, 44, 249–256. 10.1016/j.cortex.2006.05.005 18387555

[c54] Watson D., Clark L. A., & Tellegen A. (1988). Development and validation of brief measures of positive and negative affect: The PANAS scales. Journal of Personality and Social Psychology, 54, 1063–1070. 10.1037/0022-3514.54.6.1063 3397865

[c55] Wickham H. (2009). ggplot2: Elegant graphics for data analysis. New York, NY: Springer-Verlag.

[c56] Williams M. A., Moss S. A., Bradshaw J. L., & Mattingley J. B. (2005). Look at me, I’m smiling: Visual search for threatening and nonthreatening facial expressions. Visual Cognition, 12, 29–50. 10.1080/13506280444000193

[c57] Wood J. N. (2011). When do spatial and visual working memory interact? Attention, Perception, & Psychophysics, 73, 420–439. 10.3758/s13414-010-0048-8 21264717

[c58] Yonelinas A. P. (2013). The hippocampus supports high-resolution binding in the service of perception, working memory and long-term memory. Behavioural Brain Research, 254, 34–44. 10.1016/j.bbr.2013.05.030 23721964 PMC3773061

[c59] Zhang W., & Luck S. J. (2008). Discrete fixed-resolution representations in visual working memory. Nature, 453, 233–235. 10.1038/nature06860 18385672 PMC2588137

