# Supplementary material for: Remembering Who Was Where: A Happy Expression Advantage for Face Identity-Location Binding in Working Memory
Source: J Exp Psychol Learn Mem Cogn. 2018 Apr 19;44(9):1365–83. doi: 10.1037/xlm0000522 (PMC6116890; doi:10.1037/xlm0000522)
Supplement: Supplementary file 1 [file SUPP_Material-Final.docx]

# **Supplemetary Material: Model structures**

**Models Experiment 1, Working Memory performance (models 1)**

**Model 1.1.1 (Accuracy)**

*Accuracy ~ Emotion * Load +* (1 *+ Emotion * Load ||Participant*) + (1 *+ Emotion * Load ||Trial*)

**Model 1.1.2 (Accuracy at load 4)**

*Accuracy ~ Emotion +* (1 *+ Emotion ||Participant*) + (1 *+ Emotion ||Trial*)

**Model 1.2.1 (Precision)**

*Log(Distance in degrees from ROI’s centre) ~ Emotion * Load +* (1 *+ Emotion * Load || Participant*) + (1 *+ Emotion ||Trial*)

**Model 1.2.2 (Precision at load 1)**

*Log(Distance in degrees from ROI’s centre) ~ Emotion +* (1 *+ Emotion || Participant*) + (1 *+ Emotion ||Trial*)

**Model 1.3 (Swap errors, proportion)**

*Type of error ~ Emotion * Load +* (1 *+ Emotion * Load ||Participant*) + (1 *+ Emotion ||Trial*)

**Model 1.4 (Precision: correct responses vs. swap errors)**

*Log(Distance in degrees from ROI’s centre) ~ Type of response* Emotion * Load +* (1 *+ Type of response * Emotion ||Participant*) + (1 *+ Type of response * Emotion ||Trial*)

**Models Experiment 1, Individual differences (models 2)**

***Considering Autism Spectrum Quotient (ASQ), total score***

**Model 2.1.1 (Accuracy)**

*Accuracy ~ Emotion * Load * ASQ total score +* (1 *+ Emotion * Load ||Participant*) + (1 *+ Emotion ||Trial*)

**Model 2.1.2 (Precision)**

*Log(Distance in degrees from ROI’s centre) ~ Emotion * Load * ASQ total score +* (1 *+ Emotion ||Participant*) + (1 *+ Emotion ||Trial*)

***Considering score at the Negative Affect Schedule (PANAS), negative score***

**Model 2.2.1 (Accuracy)**

*Accuracy ~ Emotion * Load * PANAS negative score +* (1 *+ Emotion * Load ||Participant*) + (1 *+ Emotion ||Trial*)

**Model 2.2.1 (Precision)**

*Log(Distance in degrees from ROI’s centre) ~ Emotion * Load * PANAS negative score +* (1 *+ Emotion ||Participant*) + (1 *+ Emotion ||Trial*)

***Considering score at the Negative Affect Schedule (PANAS), positive score***

**Model 2.2.3 (Accuracy)**

*Accuracy ~ Emotion * Load * PANAS positive score +* (1 *+ Emotion * Load ||Participant*) + (1 *+ Emotion ||Trial*)

**Model 2.2.4 (Precision)**

*Log(Distance in degrees from ROI’s centre) ~ Emotion * Load * PANAS positive score +* (1 *+ Emotion * Load ||Participant*) + (1 *+ Emotion ||Trial*)

***Considering Leibowitz total score***

**Model 2.3.1 (Accuracy)**

*Accuracy ~ Emotion * Load * Leibowitz total score +* (1 *+ Emotion * Load ||Participant*) + (1 *+ Emotion ||Trial*)

**Model 2.3.2 (Precision)**

*Log(Distance in degrees from ROI’s centre) ~ Emotion * Load * Leibowitz total score +* (1 *+ Emotion + Load ||Participant*) + (1 *+ Emotion ||Trial*)

**Models Experiment 2, Working Memory performance (models 3)**

**Model 3.1 (Accuracy)**

*Accuracy ~ Emotion * Maintenance interval +* (1 *+ Emotion * Maintenance interval ||Participant*) + (1 *+ Emotion + Maintenance interval ||Tria*l)

**Model 3.2 (Precision)**

*Log(Distance in degrees from ROI’s centre) ~ Emotion * Maintenance interval +* (1 *+ Emotion * Maintenance interval || Participant*) + (1 *+ Emotion ||Trial*)

**Model 3.3 (Swap errors, proportion)**

*Type of error ~ Emotion * Maintenance interval +* (1 *+ Emotion * Maintenance interval ||Participant*) + (1 *+ Emotion ||Trial*)

**Model 3.4 (Precision: correct responses vs. swap errors)**

*Log(Distance in degrees from ROI’s centre) ~ Type of response * Emotion * Maintenance interval +* (1 *+ Type of response * Emotion * Maintenance interval ||Participant*) + (1 *+ Type of response * Emotion ||Trial*)

**Models Experiment 2, Individual differences (models 4)**

***Considering Autism Spectrum Quotient (ASQ), total score***

**Model 4.1.1 (Accuracy)**

*Accuracy ~ Emotion + Maintenance interval + ASQ total score + Emotion* : *Maintenance interval + Emotion* : *ASQ total score + Maintenance interval* : *ASQ total score* (1 *+ Emotion + Maintenance interval ||Participant*) + (1 *+ Emotion ||Trial*)

**Model 4.1.2 (Precision)**

*Log(Distance in degrees from ROI’s centre) ~ Emotion + Maintenance interval + ASQ total score + Emotion* : *Maintenance interval + Emotion* : *ASQ total score + Maintenance interval* : *ASQ total score* (1 *+ Emotion + Maintenance interval ||Participant*) + (1 *+ Emotion ||Trial*)

***Considering score at the Negative Affect Schedule (PANAS), negative score***

**Model 4.2.1 (Accuracy)**

*Accuracy ~ Emotion + Maintenance interval + PANAS negative score + Emotion* : *Maintenance interval + Emotion* : *PANAS negative score + Maintenance interval* : *PANAS negative score +* (1 *+ Emotion + Maintenance interval ||Participant*) + (1 *+ Emotion ||Trial)*

**Model 4.2.2 (Precision)**

*Log(Distance in degrees from ROI’s centre) ~ Emotion + Maintenance interval + PANAS positive score + Emotion* : *Maintenance interval + Emotion* : *PANAS positive score + Maintenance interval* : *PANAS positive score +* (1 *+ Emotion + Maintenance interval ||Participant*) + (1 *+ Emotion ||Trial*)

***Considering score at the Negative Affect Schedule (PANAS), positive score***

**Model 4.2.3 (Accuracy)**

*Accuracy ~ Emotion + Maintenance interval + PANAS positive score + Emotion* : *Maintenance interval + Emotion* : *PANAS positive score + Maintenance interval* : *PANAS positive score +* (1 *+ Emotion + Maintenance interval ||Participant*) + (1 *+ Emotion ||Trial*)

**Model 4.2.4 (Precision)**

*Log(Distance in degrees from ROI’s centre) ~ Emotion + Maintenance interval + PANAS positive score + Emotion* : *Maintenance interval + Emotion* : *PANAS positive score + Maintenance interval* : *PANAS positive score +* (1 *+ Emotion ||Participant*) + (1 *+ Emotion ||Trial*)

***Considering Leibowitz total score***

**Model 4.3.1 (Accuracy)**

*Accuracy ~ Emotion + Maintenance interval + Leibowitz total score + Emotion* : *Maintenance interval + Emotion* : *Leibowitz total score + Maintenance interval* : *Leibowitz total score +* (1 *+ Emotion + Maintenance interval ||Participant*) + (1 *+ Emotion ||Trial*)

**Model 4.3.2 (Precision)**

*Log(Distance in degrees from ROI’s centre) ~ Emotion + Maintenance interval + Leibowitz total score + Emotion* : *Maintenance interval + Emotion* : *Leibowitz total score + Maintenance interval* : *Leibowitz total score +* (1 *+ Emotion + Maintenance interval ||Participant*) + (1 *+ Emotion ||Trial*)

**Models Experiment 1 at load 4, Emotion effects on oculomotor behaviour at encoding (models 5)**

**Model 5.1 (Mean fixation duration per face)**

*Log(mean fixation duration per face) ~ Emotion +* (1 *+ Emotion ||Participant*) + (1 *+ Emotion ||Trial*)

**Model 5.2 (Mean visit duration per face)**

*Log(mean visit duration per face) ~ Emotion +* (1 *+ Emotion ||Participant*) + (1 *+ Emotion ||Trial*)

**Models Experiment 2, Emotion effects on oculomotor behaviour at encoding (models 6)**

**Model 6.1.1 (Mean fixation duration per face)**

*Log(mean fixation duration per face) ~ Emotion +* (1 *+ Emotion ||Participant*) + (1 *+ Emotion ||Trial*)

**Model 6.1.2 (Mean fixation duration per face, considering accuracy as predictor)**

*Log(mean fixation duration per face) ~ Emotion * Accuracy +* (1 *+ Emotion * Accuracy||Participant*) + (1 *+ Emotion * Accuracy ||Trial*)

**Model 6.2 (Mean visit duration per face)**

*Log(mean visit duration per face) ~ Emotion +* (1 *+ Emotion ||Participant*) + (1 *+ Emotion ||Trial*)

**Models Experiment 1 at load 4, Relationship between oculomotor behaviour at encoding on the test face and WM accuracy (models 7)**

***Considering continuous eye-movement predictors (rescaled)***

**Model 7.1**

*Accuracy ~ Emotion * total fixation time on test face +* (1 *+ Emotion * total fixation time on test face ||Participant*) + (1 *+ Emotion * total fixation time on test face ||Trial*)

**Model 7.2**

*Accuracy ~ Emotion * log(mean fixation duration on test face) +* (1 *+ Emotion * log(mean fixation duration on test face) ||Participant*) + (1 *+ Emotion * log(mean fixation duration on test face) ||Trial*)

**Model 7.3**

*Accuracy ~ Emotion * log(mean visit duration on test face) +* (1 *+ Emotion * log(mean visit duration on test face) ||Participant*) + (1 *+ Emotion * log(mean visit duration on test face) ||Trial*)

***Considering selection order***

**Model 7.4**

*Accuracy ~ Emotion * Test face selection order +* (1 *+ Emotion * Test face selection order ||Participant*) + (1 *+ Emotion ||Trial*)

***Considering exit order***

**Model 7.5**

*Accuracy ~ Emotion * Test face exit order +* (1 *+ Emotion * Test face exit order ||Participant*) + (1 *+ Emotion + Test face exit order ||Trial*)

**Models Experiment 1 at load 4, Relationship between oculomotor behaviour at encoding on the test face and WM precision (models 8)**

***Considering continuous eye-movement predictors (rescaled)***

**Model 8.1**

*Log(Distance in degrees from ROI’s centre) ~ Emotion * total fixation time on test face +* (1 *+ Emotion * total fixation time on test face ||Participant*) + (1 *+ total fixation time on test face ||Trial*)

**Model 8.2**

*Log(Distance in degrees from ROI’s centre) ~ Emotion * log(mean fixation duration on test face) +* (1 *+ Emotion * log(mean fixation duration on test face) ||Participant*) + (1 *+ Emotion * log(mean fixation duration on test face) ||Trial*)

**Model 8.3**

*Log(Distance in degrees from ROI’s centre) ~ Emotion * log(mean visit duration on test face) +* (1 *+ Emotion * log(mean visit duration on test face) ||Participant*) + (1 *+ Emotion * log(mean visit duration on test face) ||Trial*)

***Considering selection order***

**Model 8.4**

*Log(Distance in degrees from ROI’s centre) ~ Emotion * Test face selection order +* (1 *+ Emotion * Test face selection order ||Participant*) + (1 *+ Emotion * Test face selection order ||Trial*)

***Considering exit order***

**Model 8.5**

*Log(Distance in degrees from ROI’s centre) ~ Emotion * Test face exit order +* (1 *+ Emotion * Test face exit order ||Participant*) + (1 *+ Emotion ||Trial*)

**Models Experiment 2, Relationship between oculomotor behaviour at encoding on the test face and WM accuracy (models 9)**

***Considering continuous eye-movement predictors (rescaled)***

**Models 9.1.1 (at maintenance interval, MI, 1s), 9.1.2 (at MI 3s), 9.1.3 (at MI 6s)**

*Accuracy ~ Emotion * total fixation time on test face +* (1 *+ Emotion * total fixation time on test face ||Participant*) + (1 *+ Emotion * total fixation time on test face ||Trial*)

**Models 9.2.1 (at MI 1s), 9.2.2 (at MI 3s), 9.2.3 (at MI 6s)**

*Accuracy ~ Emotion * log(mean fixation duration on test face) +* (1 *+ Emotion * log(mean fixation duration on test face) ||Participant*) + (1 *+ Emotion * log(mean fixation duration on test face) ||Trial*)

**Models 9.3.1 (at MI 1s), 9.3.2 (at MI 3s), 9.3.3 (at MI 6s)**

*Accuracy ~ Emotion * log(mean visit duration on test face) +* (1 *+ Emotion * log(mean visit duration on test face) ||Participant*) + (1 *+ Emotion * log(mean visit duration on test face) ||Trial*)

***Considering selection order***

**Model 9.4.1 (at MI, 1s)**

*Accuracy ~ Emotion * Test face selection order +* (1 *+ Emotion + Test face selection order ||Participant*) + (1 *+ Emotion ||Trial*)

**Model 9.4.2 (at MI 3s)**

*Accuracy ~ Emotion * Test face selection order +* (1 *+ Emotion * Test face selection order ||Participant*) + (1 *+ Emotion * Test face selection ||Trial*)

**Models 9.4.2.1 and 9.4.2.2**: follow-up models for analysing selection order effects at MI 3s, when the test face was the first selected (9.4.2.1) and when it was the last selected (9.4.2.2)

*Accuracy ~ Emotion +* (1 *+ Emotion ||Participant*) + (1 *+ Emotion ||Trial*)

**Model 9.4.3 (at MI 6s)**

*Accuracy ~ Emotion * Test face selection order +* (1 *+ Emotion * Test face selection order ||Participant*) + (1 *+ Emotion ||Trial*)

***Considering exit order***

**Models 9.5.1 (at MI 1s) and 9.5.2 (at MI 3s)**

*Accuracy ~ Emotion * Test face exit order +* (1 *+ Emotion ||Participant*) + (1 *+ Emotion ||Trial*)

**Models 9.5.2.1 and 9.5.2.2**: follow-up models for analysing exit order effects at MI 3s, when the test face was the second exited (9.5.2.1) and when it was the last exited (9.5.2.2)

*Accuracy ~ Emotion +* (1 *+ Emotion ||Participant*) + (1 *+ Emotion ||Trial*)

**Model 9.5.3 (at MI 6s)**

*Accuracy ~ Emotion * Test face exit order +* (1 *+ Emotion + Test face exit order ||Participant*) + (1 *+ Emotion ||Trial*)

**Models Experiment 2, Relationship between oculomotor behaviour at encoding on the test face and WM precision (models 10)**

***Considering continuous eye-movement predictors (rescaled)***

**Models 10.1.1 (at MI 1s) and 10.1.3 (at MI 6s)**

*Log(Distance in degrees from ROI’s centre) ~ Emotion * total fixation time on test face +* (1 *+ Emotion * total fixation time on test face ||Participant*) + (1 *+ total fixation time on test face ||Trial*)

**Model 10.1.2 (at MI 3s)**

*Log(Distance in degrees from ROI’s centre) ~ Emotion * total fixation time on test face +* (1 *+ Emotion ||Participant*) + (1 *+ total fixation time on test face ||Trial*)

**Models 10.2.1 (at MI 1s), 10.2.2 (at MI 3s) and 10.3.3 (at MI 6s)**

*Log(Distance in degrees from ROI’s centre) ~ Emotion * log(mean fixation duration on test face) +* (1 *+ Emotion * log(mean fixation duration on test face) ||Participant*) + (1 *+ Emotion * log(mean fixation duration on test face) ||Trial*)

**Models 10.3.1 (at MI 1s), 10.3.2 (at MI 3s) and 10.3.3 (at MI 6s)**

*Log(Distance in degrees from ROI’s centre) ~ Emotion * log(mean visit duration on test face) +* (1 *+ Emotion * log(mean visit duration on test face) ||Participant*) + (1 *+ Emotion * log(mean visit duration on test face) ||Trial*)

***Considering selection order***

**Model 10.4.1 (at MI 1s)**

*Log(Distance in degrees from ROI’s centre) ~ Emotion * Test face selection order +* (1 *+ Emotion * Test face selection order ||Participant*) + (1 *+ Emotion * Test face selection order ||Trial*)

**Model 10.4.2 (at MI 3s)**

*Log(Distance in degrees from ROI’s centre) ~ Emotion * Test face selection order +* (1 *+ Emotion * Test face selection order ||Participant*) + (1 *+ Emotion + Test face selection order ||Trial*)

**Model 10.4.3 (at MI 6s)**

*Log(Distance in degrees from ROI’s centre) ~ Emotion * Test face selection order +* (1 *+ Emotion * Test face selection order ||Participant*) + (1 *+ Emotion ||Trial*)

***Considering exit order***

**Model 10.5.1 (at MI 1s)**

*Log(Distance in degrees from ROI’s centre) ~ Emotion * Test face exit order +* (1 *+ Emotion * Test face exit order ||Participant*) + (1 *+ Emotion ||Trial*)

**Model 10.5.2 (at MI 3s)**

*Log(Distance in degrees from ROI’s centre) ~ Emotion * Test face exit order +* (1 *+ Emotion * Test face exit order ||Participant*) + (1 *+ Emotion ||Trial*)

**Model 10.5.3 (at MI 6s)**

*Log(Distance in degrees from ROI’s centre) ~ Emotion * Test face exit order +* (1 *+ Emotion * Test face exit order ||Participant*) + (1 *+ Emotion + Test face exit order ||Trial*)
